# Dual Physiological Barriers Bypassed by a Silk‐Based Supramolecular Protein Delivery Platform for Neuroinflammation Mitigation in Alzheimer's Disease

**DOI:** 10.1002/advs.75342

**Published:** 2026-04-20

**Authors:** Doudou Hu, Tiandong Li, Jian He, Yujian Jiang, Yeyuan Wang, Jingchen Sun

**Affiliations:** ^1^ Guangdong Engineering Technology Research Center of Sericulture College of Animal Science South China Agricultural University Guangzhou Guangdong China; ^2^ State Key Laboratory of Quality Research in Chinese Medicine Institute of Chinese Medical Sciences University of Macau Taipa Macau SAR China

**Keywords:** Alzheimer's disease, protein delivery, silk sericin

## Abstract

Delivering protein therapeutics to the brain through nanocarriers requires overcoming both the blood‐brain barrier (BBB) and intracellular lysosomal degradation. Here, we report a silk‐based supramolecular protein delivery platform that addresses these dual physiological barriers. Two phenolic compounds are grafted onto silk sericin (SS), a biocompatible and bioactive natural protein, yielding phenolic SS capable of assembling with protein cargos via supramolecular interactions. To facilitate BBB penetration, the iRGD peptide is incorporated to enable transcytosis via the bystander effect. Phenolic modification alters the ratio of amino to carboxyl groups on SS, thereby tuning its isoelectric point. In acidic lysosomes, the nanocomplex undergoes a surface charge reversal from negative to positive, promoting lysosomal escape and cytosolic release of catalase. In parallel, phenolic SS exhibits intrinsic anti‐inflammatory activity, repolarizing activated microglia toward an anti‐inflammatory phenotype. In APP/PS1 transgenic mice, systemic administration of the nanocomplex reduces oxidative stress and neuroinflammation, leading to significant improvements in cognitive function, compared to a non‐charge‐reversal control. Collectively, this strategy provides a versatile and translatable framework for engineering protein‐based nanocarriers to deliver protein therapeutics for neurodegenerative disease treatments.

## Graphical abstract

A supramolecular nanocomplex based on phenolic‐modified silk sericin is developed to deliver antioxidant enzymes across the blood–brain barrier and escape lysosomal degradation. This dual‐barrier‐crossing system reduces neuroinflammation and improves cognitive performance in Alzheimer's disease mouse model, offering a promising strategy for protein‐based therapeutics targeting the central nervous system.

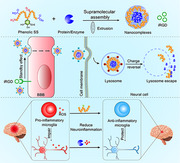

## 1 Introduction

Alzheimer's disease (AD) is a progressive neurodegenerative disorder of the central nervous system. In addition to hallmark features such as β‐amyloid (Aβ) plaques and neurofibrillary tangles, growing evidence highlights the critical roles of oxidative stress and neuroinflammation in AD pathogenesis [1, 2, 3, 4]. Antioxidant enzymes, capable of scavenging reactive oxygen species (ROS), offer a promising strategy to mitigate inflammation and neuronal damage. However, systemic delivery of these protein therapeutics remains challenging. Their poor stability and short half‐life in vivo result in rapid enzymatic deactivation. Furthermore, their therapeutic activity relies on intracellular localization, yet the hydrophilicity prevents efficient transmembrane transport [5], limiting their clinical utility.

Recently, a wide range of drug delivery systems—particularly nanoparticulate platforms—have been developed to transport protein therapeutics to disease sites [6, 7, 8, 9, 10, 11, 12]. These systems offer notable advantages, including protection against proteolytic degradation, preservation of protein activity, and prolonged circulation half‐life. However, in the context of neurodegenerative diseases, most nanocarriers face substantial barriers in crossing the blood–brain barrier (BBB) to access affected brain regions. Moreover, following cellular uptake, these carriers are often sequestered in lysosomes via endocytosis, where the encapsulated proteins may undergo enzymatic degradation and functional loss. Together, the BBB and lysosomal entrapment severely limit the therapeutic potential of protein drugs. Effective antioxidant enzyme therapies for AD therefore depend on overcoming two major challenges: enabling nanocarriers to traverse the BBB, and promoting endo/lysosomal escape within target brain cells.

Multiple strategies have been proposed to enhance the BBB permeability of nanocarriers. These include surface functionalization with targeting ligands to enable receptor‐mediated transcytosis [13, 14, 15, 16, 17, 18, 19, 20, 21, 22, 23], as well as physical methods such as focused ultrasound to transiently disrupt the BBB [24, 25, 26, 27]. However, for therapeutic proteins that require cytosolic activity, overcoming lysosomal entrapment remains equally important [28]. Typically, cationic nanocarriers are commonly employed to facilitate endo/lysosomal escape [29, 30, 31, 32], but their strong positive surface charge promotes opsonization and rapid clearance from systemic circulation. More critically, these carriers can trigger inflammatory responses [33]. Given that AD is a progressive, chronic disease requiring long‐term treatment, the biosafety of delivery systems is of paramount importance.

Silk sericin (SS), a natural biopolymer derived from silk fibers, is distinguished by its excellent biocompatibility, biodegradability, and intrinsic bioactivity [34]. SS exhibits low immunogenicity [35] and possesses multiple therapeutic properties, including anti‐inflammatory [36], hypoglycemic [37], and hypolipidemic effects [38]. In small animal models, SS has been shown to ameliorate memory impairments induced by aging and sleep deprivation [39, 40]. Beyond its biological functions, SS contains abundant polar amino acids—such as serine and asparagine—which provide accessible sites for chemical modification [34], making it a versatile platform for biomaterial engineering.

**FIGURE 1:**
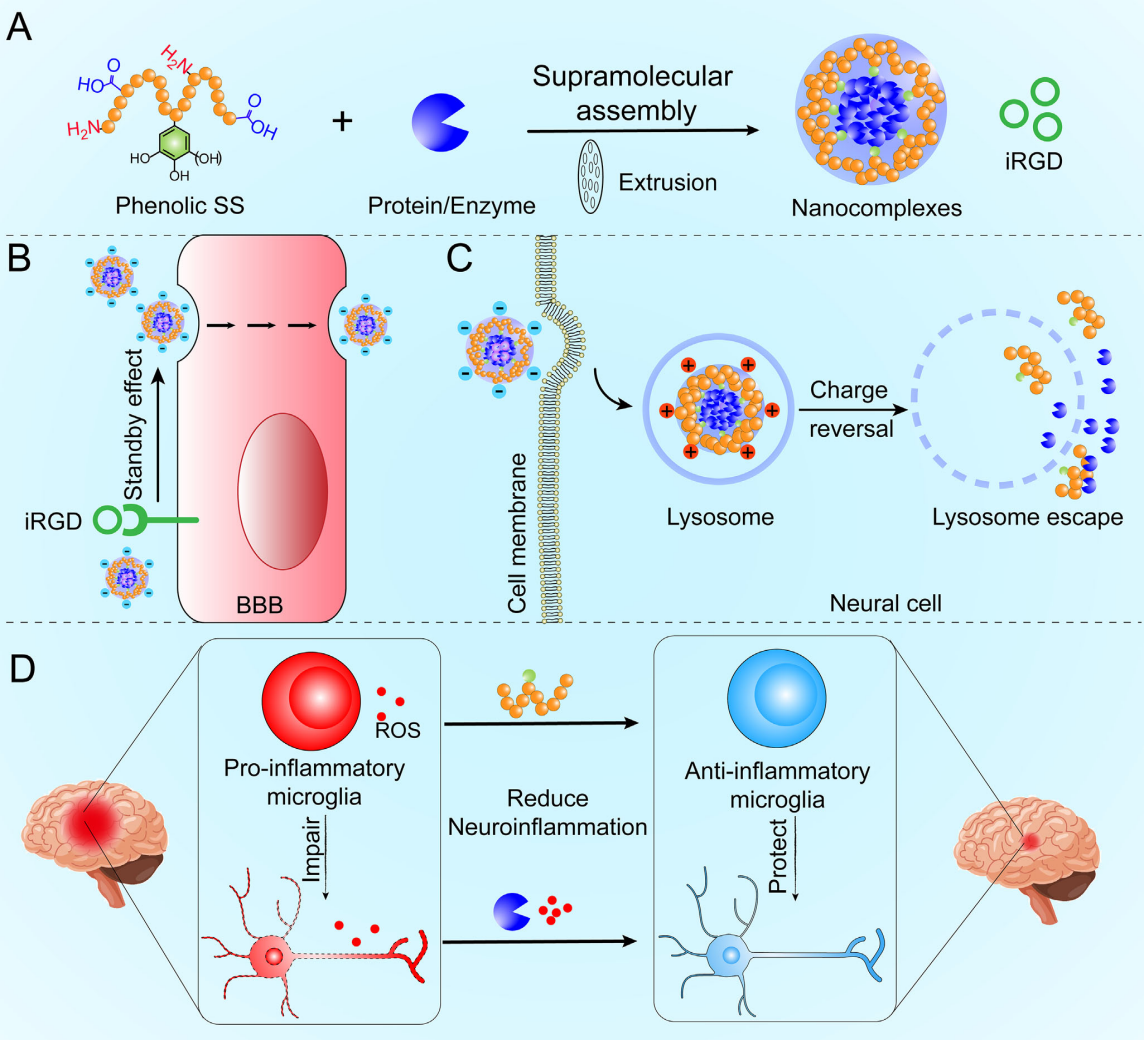
Schematic illustration of the design and function of silk‐based supramolecular nanocomplexes for brain delivery. (A) Supramolecular assembly of antioxidant enzymes with phenolic‐modified silk sericin (SS) to form nanocomplexes. (B) iRGD facilitates nanocomplex transcytosis across the blood‐brain barrier (BBB) via the bystander effect. (C) Upon internalization, the nanocomplex escapes from lysosomes through acid‐triggered surface charge reversal. (D) Delivered antioxidant enzymes and the bioactive phenolic SS synergistically mitigate neuroinflammation in Alzheimer's disease (AD).

To enable effective brain delivery of protein therapeutics, we developed a SS‐based supramolecular nanocomplex for antioxidant enzyme delivery. SS was chemically modified with polyphenolic groups, which drive supramolecular assembly with enzymes through non‐covalent interactions (Figure 1A). Then, the cyclic peptide iRGD was incorporated to the nanocomplex system to facilitate BBB penetration via fluid‐phase transcytosis (bystander effect) (Figure 1B) [41, 42]. Structurally, polyphenol modification modulates the ratio of amino to carboxyl groups on SS, tuning its isoelectric point (pI). This allows the nanocomplex to undergo a surface charge reversal from negative to positive upon exposure to the acidic lysosomal environment, promoting lysosomal escape of the protein cargo (Figure 1C). Functionally, phenolic SS exhibits intrinsic anti‐inflammatory activity, promoting microglial polarization toward an anti‐inflammatory phenotype and synergizing with the delivered antioxidant enzymes to mitigate neuroinflammation in a AD mouse model (Figure 1D). Altogether, this work presents a versatile and biocompatible delivery platform capable of overcoming dual physiological barriers, offering a broadly applicable strategy for the intracellular delivery of protein therapeutics to the brain.

## 2 Results

### 2.1 Preparation, Characterization, and Bioactivity of SSDA/SSGA

**FIGURE 2:**
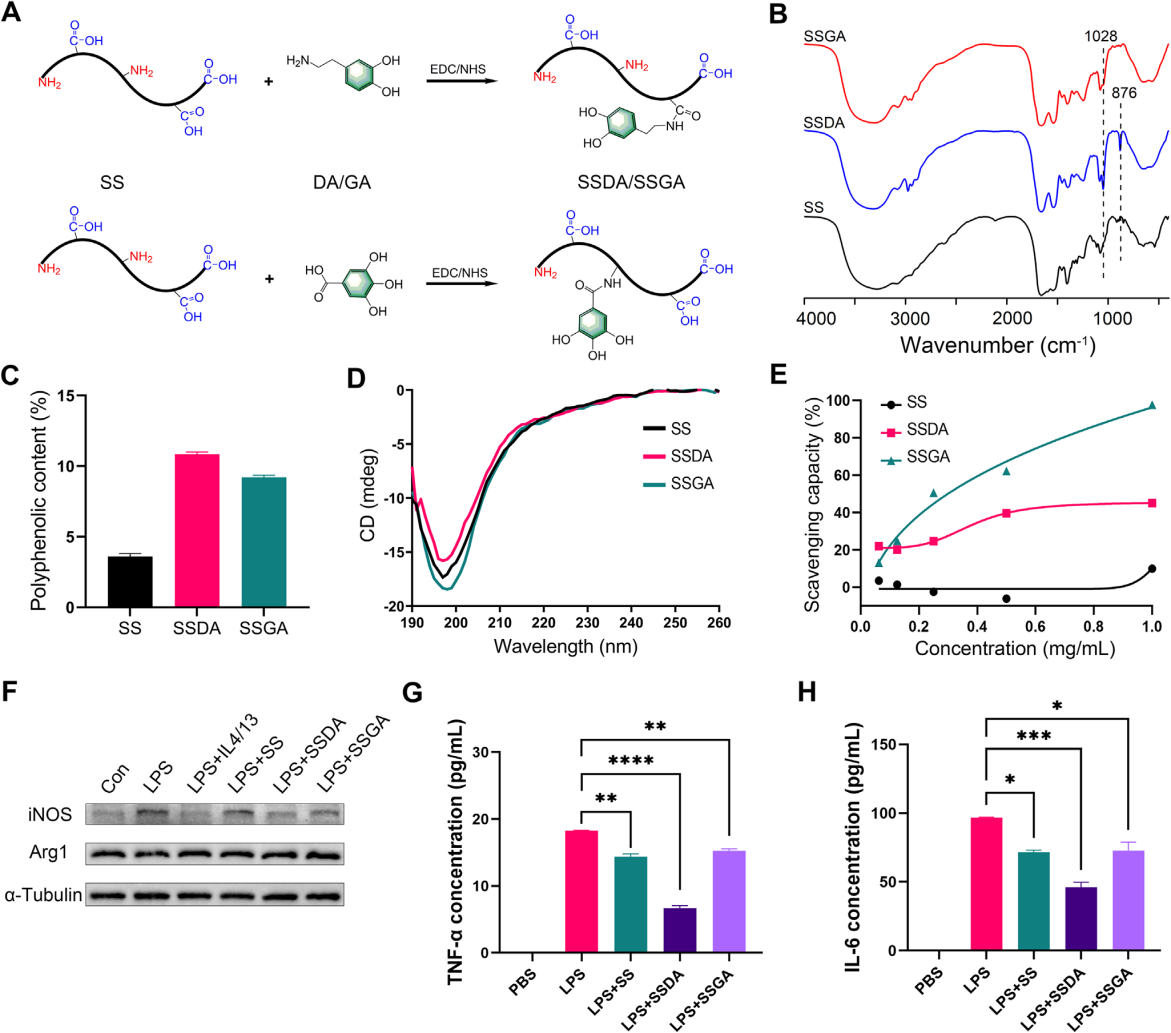
Preparation, characterization, and bioactivity of phenolic‐modified silk sericin (SSDA and SSGA). (A) Synthetic route for dopamine (DA)‐ and gallic acid (GA)‐modified silk sericin (SSDA and SSGA). (B) FT‐IR spectra of SS, SSDA, and SSGA. (C) Quantification of polyphenol content in SSDA and SSGA using the Folin‐Ciocalteu assay. (D) Circular dichroism spectra of SS, SSDA, and SSGA. (E) DPPH radical scavenging activity of SS, SSDA, and SSGA. (F) Western blot analysis of iNOS and Arg1 expression in LPS‐stimulated Raw264.7 cells treated with SS, SSDA, or SSGA for 24 h. (G, H) Quantification of pro‐inflammatory cytokines TNF‐α (G) and IL‐6 (H) in the culture supernatants after 24 h of treatment. Data represent mean ± SD (n = 3); *P < 0.05, **P < 0.01, ***P < 0.001, ****P < 0.0001.

To synthesize phenolic SS, we conjugated dopamine (DA) and gallic acid (GA) onto SS via EDC‐mediated amide coupling (Figure 2A). Fourier‐transform infrared (FT‐IR) spectra confirmed successful modification, with characteristic peaks of DA and GA observed in SSDA and SSGA, respectively (Figure 2B; Figure S1). Quantification of polyphenol content revealed DA and GA incorporation levels of 10.8% and 9.2%, respectively (Figure 2C). Circular dichroism (CD) spectra showed that SSDA, SSGA, and unmodified SS all exhibited a prominent negative peak at 200–220 nm, consistent with a random coil secondary structure (Figure 2D), indicating that phenolic modification did not significantly alter the protein's conformation. The antioxidant properties of the phenolic SS were assessed using a DPPH radical scavenging assay. Both SSDA and SSGA displayed enhanced DPPH scavenging activity compared to native SS (Figure 2E), confirming that phenolic modification imparts improved antioxidant capacity.

SSDA and SSGA exhibited excellent cytocompatibility, comparable to unmodified SS, even at concentrations up to 1000 µg/mL (Figure S2). We next evaluated their antioxidant and anti‐inflammatory effects in vitro using lipopolysaccharide (LPS)‐stimulated Raw264.7 cells. Both SSDA and SSGA significantly reduced LPS‐induced ROS production, as assessed by confocal laser scanning microscopy (CLSM) and flow cytometry (Figure S3). Macrophage polarization was further examined by western blot analysis. Compared with LPS treatment alone, SS reduced expression of inducible nitric oxide synthase (iNOS), a marker of pro‐inflammatory (M1) macrophages, while both SSDA and SSGA led to an even greater suppression of iNOS expression (Figure 2F). Meanwhile, pro‐inflammatory cytokines including TNF‐α and IL‐6 were downregulated in the presence of SS, SSDA, and SSGA, with SSDA showing the strongest effect (Figure 2G,H).

### 2.2 Construction of Protein Delivery Platform via SSDA/SSGA

**FIGURE 3:**
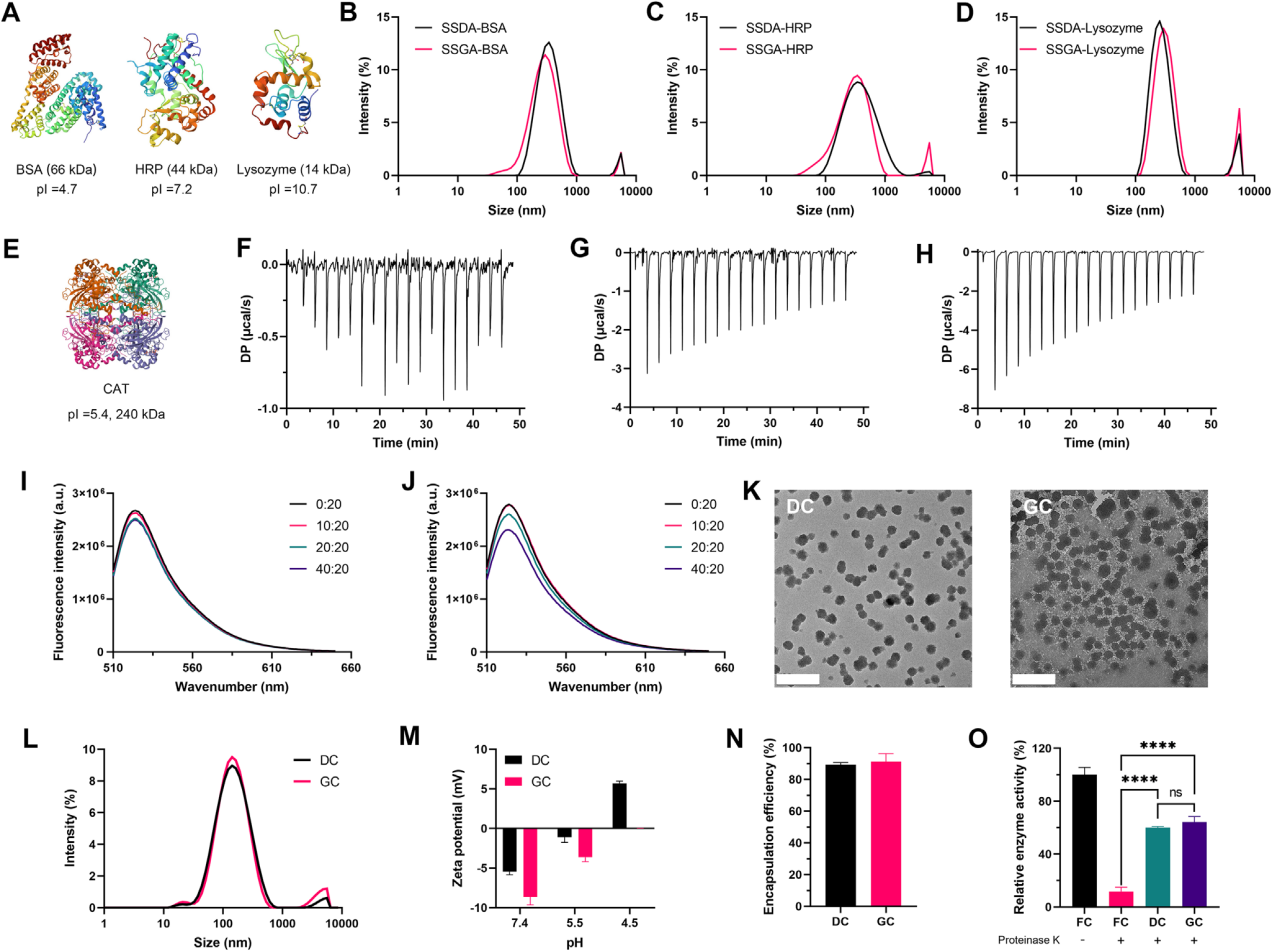
Construction and characterization of SSDA/SSGA‐based nanocomplexes. (A) Model proteins with varying isoelectric points (pIs) and molecular weights used for evaluating complexation. (B‐D) Size distributions of SSDA/SSGA‐based nanocomplexes determined by dynamic light scattering (DLS). (E) Schematic representation of catalase (CAT) as a therapeutic model protein. (F‐H) Representative isothermal titration calorimetry (ITC) curves showing interactions of CAT with SS (F), SSDA (G), and SSGA (H). (I‐J) Fluorescence quenching of FITC‐labeled CAT (^FITC^CAT) upon titration with SSDA (I) and SSGA (J). λ_ex_ = 488 nm. (K) Transmission electron microscopy (TEM) images of SSDA‐CAT (DC) and SSGA‐CAT (GC) nanocomplexes. Scale bar, 500 nm. (L) Size distribution of DC and GC determined by DLS. (M) pH‐dependent zeta potential profiles of DC and GC. (N) Encapsulation efficiency of CAT in DC and GC. (O) Relative CAT activity in FC, DC, and GC incubated with proteinase K (0.025 mg/mL) at 37°C for 4 h. Data represent mean ± SD (n = 3); ****P < 0.0001, ns, not significant.

To establish a universal protein delivery platform, we selected three model proteins with distinct isoelectric points (pIs) and molecular weights: bovine serum albumin (BSA), horseradish peroxidase (HRP), and lysozyme (Figure 3A). Simple mixing and homogenization of SSDA or SSGA with these proteins yielded nanoparticles ranging from approximately 200 to400 nm, as measured by dynamic light scattering (DLS) and transmission electron microscopy (TEM) (Figure 3B–D; Figure S4). In contrast, the hydrodynamic diameters of the free proteins were all below 10 nm (Figure S5), indicating that SSDA and SSGA effectively form supramolecular complexes with diverse protein cargos.

Catalase (CAT), an antioxidant enzyme that catalyzes the decomposition of hydrogen peroxide, was then chosen as a model therapeutic protein for further investigation (Figure 3E). Isothermal titration calorimetry (ITC) confirmed supramolecular interactions between CAT and phenolic SS. While native SS exhibited negligible binding to CAT, both SSDA and SSGA displayed clear binding affinities, with association constants (Ka) of (1.67 ± 0.029) ×10^4^ m ^−1^, and (3.95 ± 0.012) ×10^4^ m ^−1^, respectively (Figure 3F–H; Figure S6). To further validate this interaction, CAT was fluorescently labeled with FITC. Upon gradual addition of SSDA or SSGA, the fluorescence intensity of ^FITC^CAT progressively decreased (Figure 3I,J), likely due to aggregation‐induced quenching resulting from nanocomplex formation. To investigate how non‐covalent binding (ionic interactions, hydrogen bonding, and/or hydrophobic interactions) governs the formation of nanocomplexes, sodium chloride (NaCl), urea, or Tween 20 was added to the nanocomplexes to disrupt these interactions. Urea and Tween 20, which disrupt hydrogen bonds and hydrophobic interactions, respectively, reversed the decline of fluorescence intensity of FITC following ^FITC^DC and ^FITC^GC formation (Figure S7), indicating these two interactions dominate nanocomplex formation. Together, these data indicate that polyphenol modification enhances the non‐covalent interactions between SS and protein cargos, enabling stable nanocomplex formation.

The morphology of two nanocomplexes, SSDA‐CAT (DC) and SSGA‐CAT (GC), was visualized by TEM, revealing uniformly spherical particles (Figure 3K). DLS analysis showed that both DC and GC had average diameters of approximately 190 nm, with polydispersity index (PDI) values of 0.370 and 0.371, respectively (Figure 3L). These nanocomplexes remained stable in phosphate‐buffered saline (PBS) over the course of one week (Figure S8). Given that DA and GA conjugation differentially alters the ratio of carboxyl to amino groups in SS (Figure 1A), the resulting pI values of SSDA and SSGA also differed (Figure S9). To assess charge‐reversal behavior, we measured the zeta potential of DC and GC under varying pH conditions. DC exhibited a clear surface charge transition from negative to positive as the pH decreased, whereas GC retained a negative surface charge across the tested pH range (Figure 3M). These results suggest that only DC is capable of lysosomal escape via pH‐responsive charge reversal. The average encapsulation efficiency of CAT in DC and GC were 89.29% and 91.12%, respectively (Figure 3N). The CAT release profiles of both DC and GC under different pH values were not apparent (Figure S10). This phenomenon is possibly attributed to the fact that the dominant interactions between SSDA/SSGA and CAT are hydrogen bonding and hydrophobic interactions; therefore, ionic interactions induced by pH variations have a negligible effect on nanocomplex disassembly. It implies that ionic/electrostatic interactions do not mediate the nanocomplex formation. To determine whether complexation with SSDA or SSGA affects enzymatic activity, we compared the catalytic function of encapsulated CAT with that of free CAT (FC). No significant loss of enzyme activity was observed in either DC or GC (Figure S11), indicating that the supramolecular assembly process preserves CAT functionality. We evaluated the stability of CAT by measuring its enzymatic activity in the presence of proteinase K. FC was rapidly degraded and lost its activity upon exposure to proteinase K. In contrast, both DC and GC effectively protected the complexed CAT from proteolytic inactivation (Figure 3O).

### 2.3 Lysosome Escape Investigation and Therapeutic Effect of DC and GC

**FIGURE 4:**
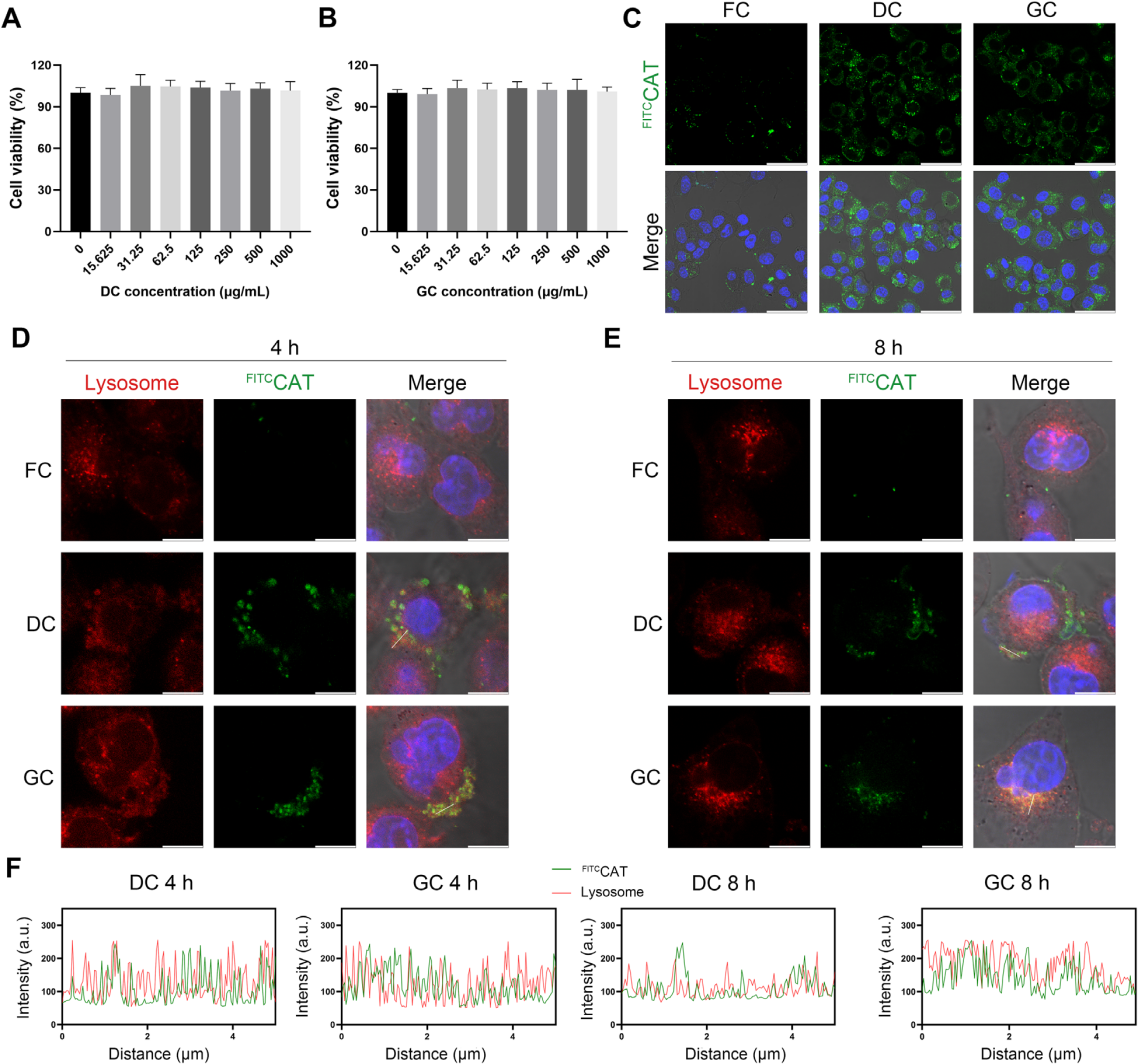
Intracellular uptake and lysosome escape ability of CAT delivered by DC and GC. (A, B) Cell biocompatibility of DC and GC on N2a cells after 24 h incubation. Data represent mean ± SD (n = 5). (C) Confocal laser scanning microscopy (CLSM) images of N2a cells after 4 h incubation with free CAT (FC), DC, or GC. CAT was labeled with FITC. Scale bar, 20 µm. (D,E) CLSM images showing co‐localization of ^FITC^CAT (green) with lysosomes (red, LysoTracker) in BV2 cells following 4 h (D) and 8 h (E) of incubation. (F) Fluorescence intensity profiles along selected lines in (D) and (E).

We first assessed the cytocompatibility of DC and GC nanocomplexes in N2a cells. As shown in Figure 4A,B, neither formulation affected cell viability, even at concentrations up to 1000 µg/mL. To evaluate cellular uptake, CAT was labelled with FITC. In cells treated with FC, minimal fluorescence was observed and primarily localized to the cell membrane, indicating poor membrane permeability. In contrast, cells treated with DC or GC exhibited strong cytoplasmic fluorescence, demonstrating efficient internalization of CAT via the nanocomplexes (Figure 4C). Lysosomes represent a major intracellular barrier for nanoparticle‐based protein delivery. To examine whether DC and GC enable cytosolic delivery of CAT in a pH‐dependent manner, we performed colocalization studies using LysoTracker Red to label lysosomes to track intracellular distribution of ^FITC^CAT in BV2 cells. In the FC group, green fluorescence was barely detectable, consistent with negligible cellular uptake. In GC‐treated cells, ^FITC^CAT signals remained co‐localized with lysosomes throughout the time course. In contrast, in DC‐treated cells, only partial colocalization was observed at 4 h, and by 8 h, most ^FITC^CAT fluorescence was separated from lysosomal staining (Figure 4D–F), indicating successful lysosomal escape. A similar pattern was also observed in N2a cells (Figure S12). These results confirm that DC enables efficient cytosolic delivery of CAT through pH‐triggered charge reversal, overcoming lysosomal entrapment.

**FIGURE 5:**
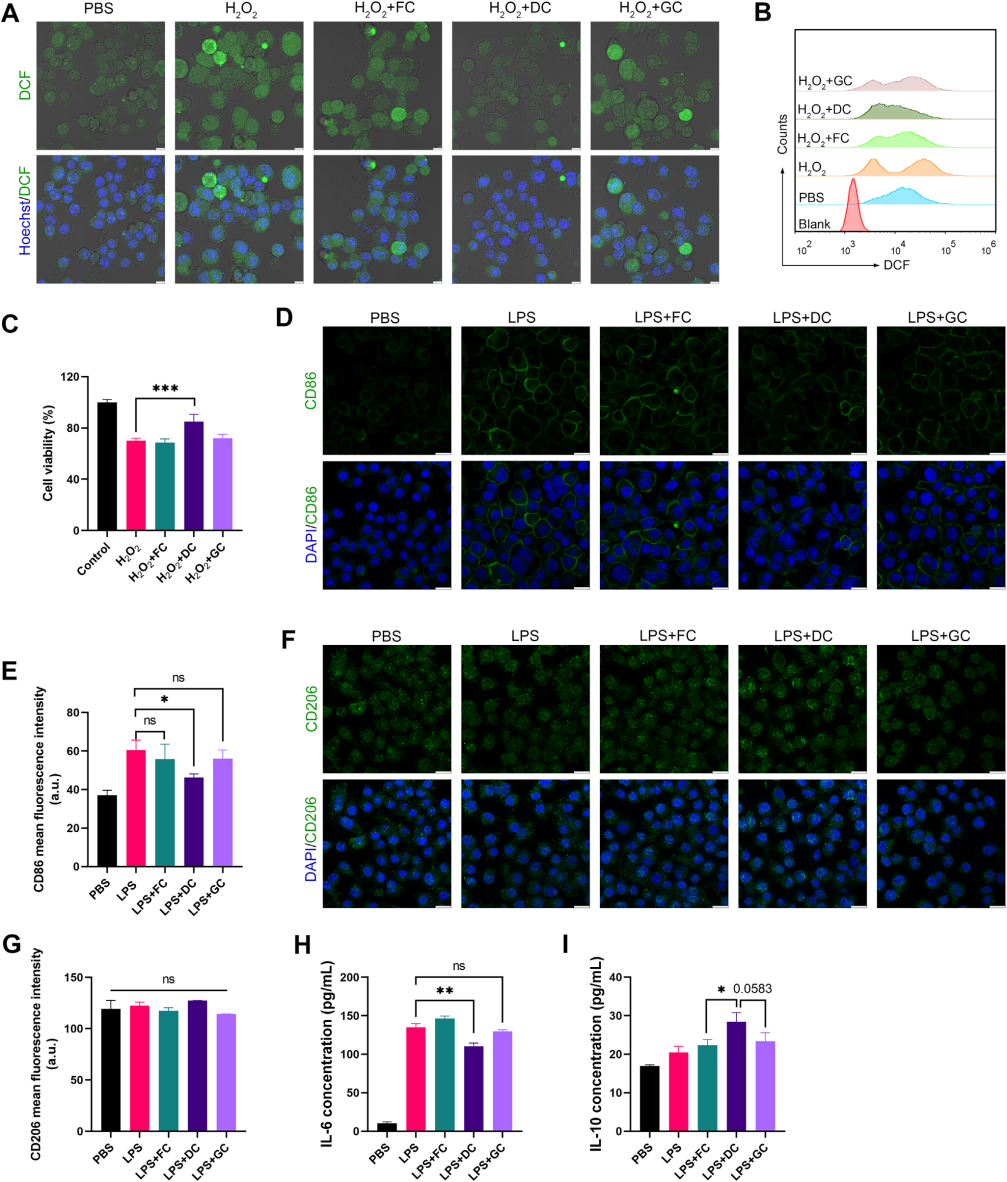
Anti‐oxidation and anti‐inflammation effects of DC and GC nanocomplexes. (A,B) Intracellular ROS levels in H_2_O_2_‐treated N2a cells following 24 h incubation with various formulations, visualized by DCFH‐DA staining using CLSM (A) and quantified by flow cytometry (B). Scale bar, 10 µm. (C) Cell viability of DC and GC on N2a cells following incubation of nanocomplexes for 24 h in the presence of H_2_O_2_. (D,E) Immunofluorescence images (D) and quantification (E) of CD86 expression (M1 microglial marker) in LPS‐stimulated BV2 cells following treatment for 24 h. Scale bar, 20 µm. (F,G) Immunofluorescence images (F) and quantification (G) of CD206 expression (M2 microglial marker) in treated BV2 cells. Scale bar, 20 µm. (H,I) ELISA quantification of IL‐6 (H) and IL‐10 (I) levels in BV2 cell supernatants following different treatments. Data represent mean ± SD (n = 3); *P < 0.05, **P < 0.01, ***P < 0.001.

We next evaluated the therapeutic potential of CAT‐loaded nanocomplexes in an in vitro oxidative stress model. Intracellular ROS levels were assessed in H_2_O_2_‐treated N2a cells. Compared with FC, DC‐treated cells exhibited significantly reduced ROS accumulation, as indicated by diminished green fluorescence intensity, whereas GC‐treated cells showed minimal improvement (Figure 5A). These results were further validated by flow cytometry (Figure 5B; Figure S13). To assess neuroprotection, we examined cell viability in N2a cells subjected to H_2_O_2_‐induced oxidative stress. As shown in Figure 5C, H_2_O_2_ exposure led to substantial cytotoxicity. Only the DC group was able to reverse the oxidative damage and restore cell viability, while FC and GC treatments offered no protective effect. These findings suggest that DC, with its lysosomal escape capability, confers superior neuroprotective efficacy. We further investigated the anti‐inflammatory effects of the nanocomplexes in LPS‐stimulated BV2 microglial cells. LPS treatment markedly increased the expression of CD86, a classical M1 microglial surface marker. Neither FC nor GC reduced CD86 expression, whereas DC treatment significantly suppressed CD86 levels (*P *< 0.05) (Figure 5D,E). No significant changes were observed in the expression of CD206, an M2 polarization marker (*P *> 0.05) (Figure 5F,G). Cytokine levels in the culture supernatants were also analyzed. Compared with FC, DC treatment reduced IL‐6 secretion by 25% (Figure 5H), while increasing IL‐10 production by 1.27‐fold (Figure 5I). To differentiate the bioactive effect of CAT and its carrier, we investigated the level of CD86 expression in LPS‐stimulated BV2 cells by comparing nanocomplexes encapsulating CAT or BSA. CD86 expression was significantly lower in the DC group than in SSDA‐BSA nanocomplex (DB) group (*P *< 0.05) (Figure S14), implying that CAT can be efficiently delivered by DC outside of lysosomes to function. Collectively, these results demonstrate that DC, but not GC, effectively mitigates oxidative stress and LPS‐induced neuroinflammation, highlighting the importance of lysosomal escape in therapeutic efficacy.

### 2.4 BBB Penetration and In Vivo Distribution of the Nanocomplexes

**FIGURE 6:**
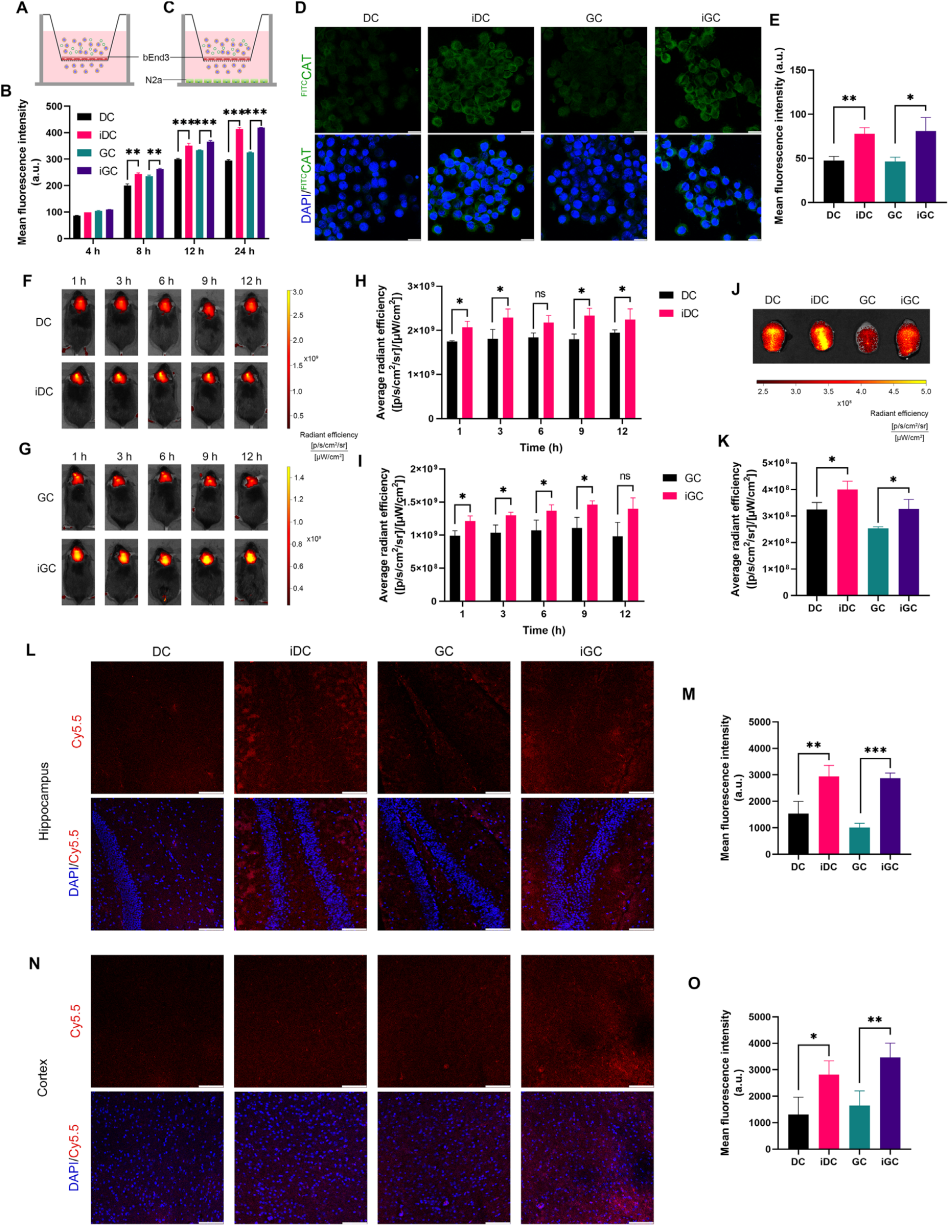
In vitro BBB penetration and in vivo distribution of the nanocomplexes. (A) Schematic of the in vitro Transwell model with bEnd.3 cells cultured on the upper chamber mimic the BBB. (B) Fluorescence intensity of the ^FITC^CAT in the lower chamber at indicated time points. (C) Schematic of in vitro Transwell model with N2a cells seeded in the lower chamber. (D) Representative CLSM images of the intracellular ^FITC^CAT accumulation in N2a cells after 24 h treatment. Scale bar, 20 µm. (E) Quantification of FITC fluorescence intensity in N2a cells from (D). (F,G) In vivo fluorescence imaging of C57BL/6 mice at indicated time points following intravenous administration of Cy5.5‐labeled nanocomplexes (n = 3). (H,I) Semiquantitative analysis of brain fluorescence intensity from (F) and (G), respectively. (J) Ex vivo fluorescence imaging of excised brains 12 h post‐injection. (K) Quantification of brain fluorescence intensities from (J). (L,M) Distribution of Cy5.5 labeled nanocomplexes in the hippocampus and the quantification of fluorescence intensity of Cy5.5. (N,O) Distribution of Cy5.5 labeled nanocomplexes in the cortex and the quantification of fluorescence intensity of Cy5.5. Data represent mean ± SD (n = 3); *P < 0.05, **P < 0.01, ***P < 0.001.

BBB poses a major obstacle to the delivery of therapeutics to the brain. To enhance BBB permeability, we functionalized the nanocomplexes with iRGD, a cyclic peptide known to mediate transcytosis across the BBB. The in vitro BBB‐penetrating ability of iRGD‐mixed nanocomplexes—iRGD + DC (iDC) and iRGD + GC (iGC)—was evaluated using a Transwell model. Transepithelial electrical resistance (TEER) value of bEnd.3 cells following 7 day culture indicated the formation of cell monolayer (Figure S15). bEnd.3 cells were seeded on the upper chamber and incubated with FITC‐labeled nanocomplexes, while the fluorescence intensity in the lower chamber medium was measured to assess transcytosis efficiency (Figure 6A). Compared to bare DC and GC, iDC, and iGC displayed significantly higher FITC fluorescence in the lower chamber after 8 h of incubation, indicating enhanced BBB penetration (Figure 6B). To further assess functional delivery across the endothelial barrier, N2a cells were cultured in the lower chamber of the Transwell system (Figure 6C). CLSM revealed markedly stronger intracellular FITC fluorescence in N2a cells treated with iDC and iGC, compared to those treated with unmodified DC and GC (Figure 6D,E). These findings indicate that iRGD facilitates the transcytosis and subsequent neuronal uptake of nanocomplexes.

To determine whether iRGD enhances brain accumulation of the nanocomplexes in vivo, DC and GC were labeled with the near‐infrared dye Cy5.5. Following systemic administration in C57BL/6 mice, fluorescence imaging revealed that iDC‐treated animals exhibited stronger brain fluorescence signals compared to those receiving DC (Figure 6F). A similar enhancement was observed in the iGC group relative to GC (Figure 6G). Quantitative analysis confirmed significantly increased brain accumulation of both iDC and iGC compared to their respective controls (*P *< 0.05) (Figure 6H,I), suggesting that iRGD markedly improves nanocomplex biodistribution to the brain. Ex vivo fluorescence imaging of major organs 12 h post‐injection further supported these findings. Fluorescence intensity in the brain was significantly higher in the iDC and iGC groups than in the DC and GC groups, respectively (*P *< 0.05) (Figure 6J,K), consistent with the in vivo data. Notably, liver and kidney fluorescence signals were reduced in the iDC and iGC groups compared to DC and GC, suggesting a favorable shift in biodistribution (Figure S16). To further assess brain localization, brain sections were analyzed by confocal microscopy. In both the hippocampus (Figure 6L,M) and cortex (Figure 6N,O), iDC and iGC produced significantly stronger fluorescence signals than their non–iRGD‐modified counterparts, confirming that iRGD effectively enhances BBB permeability and facilitates nanocomplex delivery into the brain. As the “bystander effect” of iRGD peptide is a three‐step process involving integrin binding, proteolytic cleavage, NRP1 engagement, we investigated the preliminary mechanism of this effect focusing on NRP1 in the context of APP/PS1 mice. Immunofluorescence slices demonstrated the expression of both integrin αV and NRP1 in brain of APP/PS1 mice (Figure S17). By intravenous injection of NRP1 blocking antibody (αNRP1), we again examined the brain distribution of ^Cy5.5^iDC in APP/PS1 mice. NRP1 blocking can significantly decrease the brain accumulation of fluorescence signal 6 h post administration (Figure S18), showing that NRP1 plays a role in iRGD‐mediated nanoparticle transport in the context of AD.

### 2.5 In Vivo Therapeutic Effects of Nanocomplexes in APP/PS1 Transgenic Mice

**FIGURE 7:**
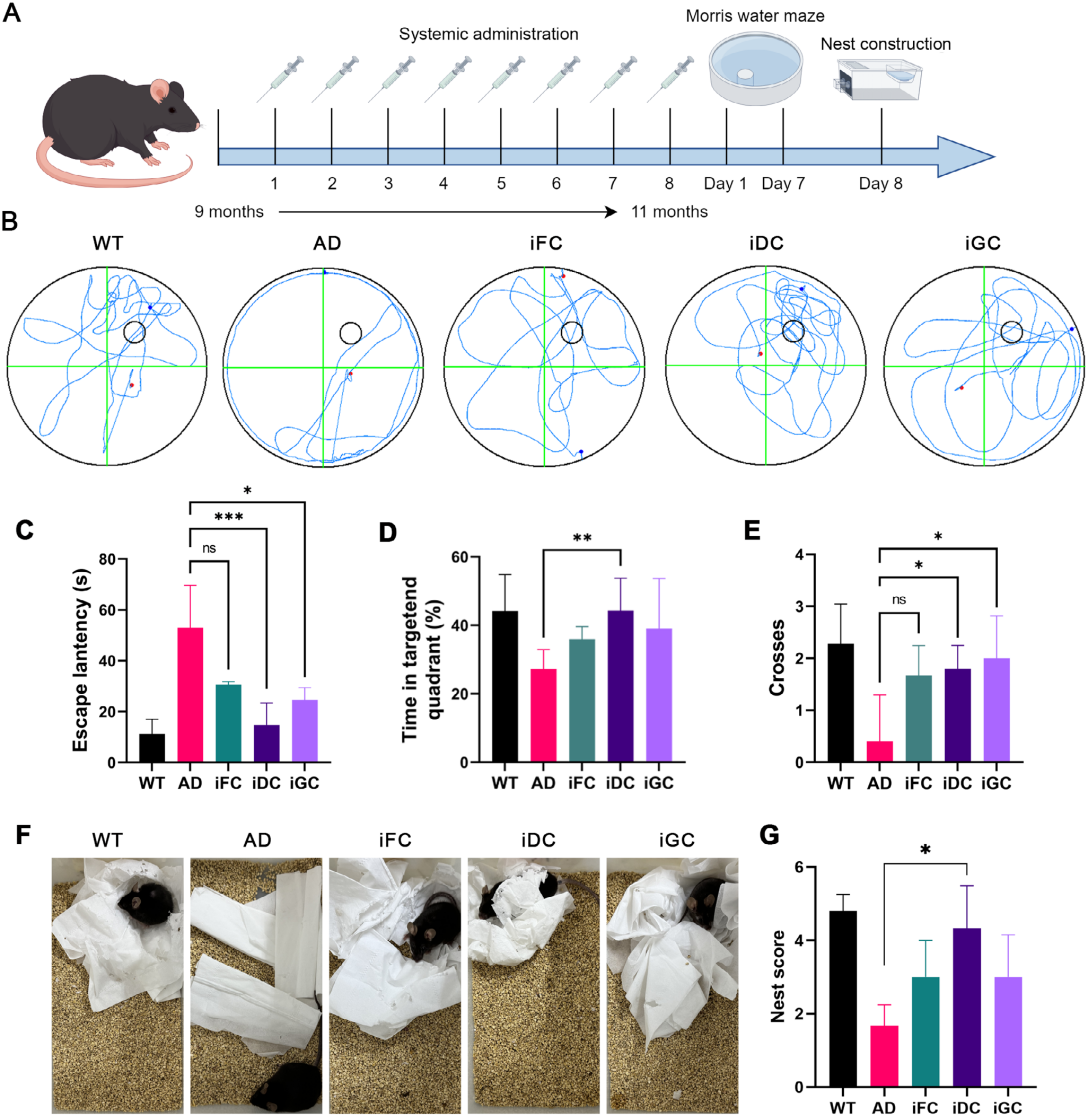
Rescue of cognitive and memory deficits by nanocomplexes in APP/PS1 transgenic mice. (A) Schematic timeline of systemic drug administration and behavioral evaluation. (B) Representative swim path tracings from the Morris water maze test. (C‐E) Quantitative analysis of escape latency (C), time spent in the target quadrant (D), and number of platform crossings (E) in wild‐type (WT) and APP/PS1 mice following various treatments. (F) Representative images of nest construction behavior in each group. (G) Scores for nest construction. Data represent mean ± SD (n = 5); *P < 0.05, **P < 0.01, ***P < 0.001.

Finally, we evaluated the in vivo therapeutic efficacy of the nanocomplexes in APP/PS1 transgenic mice using the workflow illustrated in Figure 7A. Mice were systemically administered iRGD‐enhanced formulations—iFC (iRGD + free CAT), iDC, and iGC—for about two months. Wild‐type (WT) C57BL/6 mice and APP/PS1 mice treated with PBS served as positive and negative controls, respectively. Cognitive function was assessed using the Morris water maze (MWM) test. After four days of training, the swimming trajectories to the hidden platform were recorded using a video tracking system (Figure 7B). During the probe test, PBS‐treated APP/PS1 mice exhibited significantly longer escape latency than WT mice, indicative of impaired spatial memory. Although a reduction in escape latency was observed in the iFC group, the difference was not statistically significant (*P *> 0.05). In contrast, mice in the iDC group showed a marked improvement in learning and memory, with significantly shortened escape latency (*P *< 0.001), while the iGC group also showed improvement (*P *< 0.05) (Figure 7C). Additional cognitive indicators, including time spent in the target quadrant and the number of platform‐site crossovers, were significantly enhanced in the iDC group compared to PBS‐treated APP/PS1 mice (Figure 7D,E), confirming improved memory retention. To further assess sensorimotor and motivational behavior, the nest construction test was performed. WT mice built compact and insulated nests, whereas APP/PS1 mice constructed poorly organized nests with low structural integrity. Treatment with iDC significantly improved nest‐building performance, as reflected by higher nesting scores (*P *< 0.05) (Figure 7F,G). These behavioral outcomes demonstrate that iDC treatment effectively alleviates cognitive and behavioral deficits in APP/PS1 mice.

**FIGURE 8:**
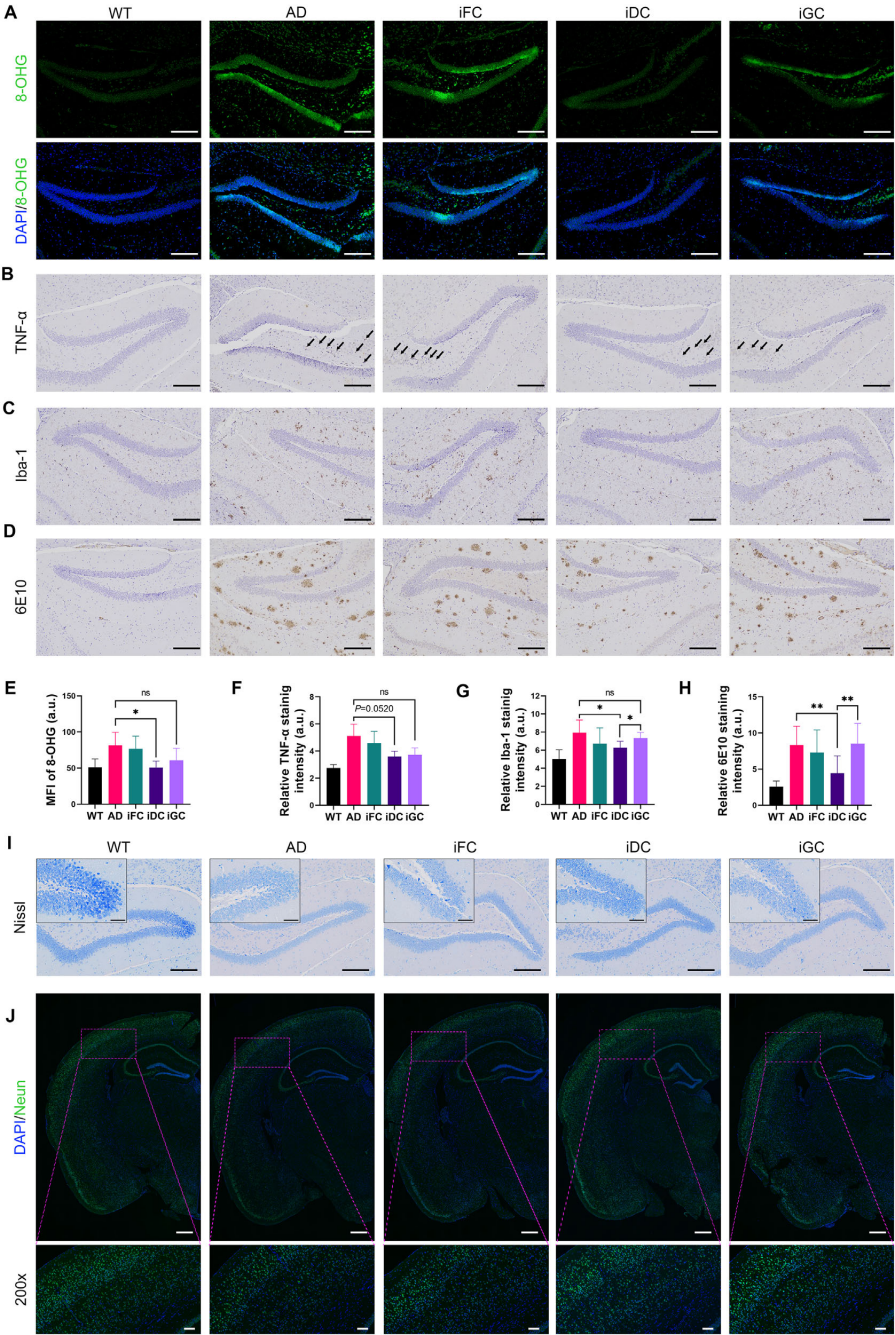
In vivo evaluation of nanocomplexes on oxidative stress, neuroinflammation, microglial activation, Aβ deposition, and neuronal protection in APP/PS1 mice. (A, E) Immunofluorescence staining of 8‐hydroxy‐2′‐deoxyguanosine (8‐OHG) in the hippocampus and corresponding quantification of oxidative damage. Scale bar, 200 µm. (B,F) Immunohistochemical staining and quantification of TNF‐α expression in the hippocampus. Scale bar, 200 µm. (C,G) Immunohistochemical staining of microglia labeled with Iba‐1 in the hippocampus and quantification of microglial reactivity. Scale bar, 200 µm. (D,H) Immunohistochemical staining and quantification of Aβ plaques using the 6E10 antibody. Scale bar, 200 µm. (I) Nissl staining of hippocampal neurons. Scale bar, 200 µm; inset, 50 µm. (J) Immunofluorescence staining of NeuN in the cortex. Scale bar, 500 µm; inset, 200 µm. Data represent mean ± SD (n = 3); *P < 0.05, **P < 0.01.

To assess pathological changes following treatment, mouse brains were harvested for immunostaining analyses. The level of 2’‐deoxy‐8‐oxoguanosine (8‐OHG), a biomarker indicative of oxidative damage, was measured to evaluate the ROS level in brain tissue by immunofluorescence assay. As shown in Figure 8A,E, 8‐OHG levels in the hippocampus were significantly reduced in the iDC group (*P *< 0.05), whereas no significant changes were observed in the iFC group (*P *> 0.05). The iGC group exhibited a downward trend, though not statistically significant (*P *> 0.05). Proinflammatory cytokine production was assessed by TNF‐α staining in the hippocampus. TNF‐α levels declined modestly in both iDC and iGC groups, although the reductions did not reach statistical significance (Figure 8B,F). Microglial activation was evaluated using Iba‐1 immunostaining. Compared to PBS‐treated APP/PS1 mice, the iDC group showed a significant reduction in Iba‐1 fluorescence intensity (*P *< 0.05), which was also significantly lower than that observed in the iGC group (*P *< 0.05) (Figure 8C,G). Amyloid pathology was analyzed by 6E10 immunostaining. The iDC group exhibited significantly fewer amyloid plaques than both the PBS‐treated APP/PS1 mice and the iGC group (Figure 8D,H). We evaluated the neuroprotective effect of different formulations by Nissl and Neun staining. In WT mice, healthy hippocampal neurons exhibited dense Nissl granules and distinct morphological boundaries, while APP/PS1 mice showed sparse granules and poor neuronal structure (Figure 8I). Only iDC treatment restored staining intensity of Nissl granules. NeuN (neuronal nuclei) staining, a marker of mature neurons, revealed reduced fluorescence in the cortex of APP/PS1 mice. Following treatment, both iDC and iGC groups showed increased NeuN fluorescence, with more pronounced improvement in the iDC group (Figure 8J). Last, preliminary biosafety was assessed via histological examination of major organs. No significant body weight loss of mice was observed (Figure S19). No significant pathological changes were observed in the heart, liver, spleen, lung, or kidney in either the iDC or iGC groups after treatment (Figure S20), supporting the biocompatibility of the nanocomplexes.

## 3 Discussion

Efficient delivery of protein therapeutics to the brain remains one of the most formidable challenges in the treatment of neurodegenerative diseases. In this study, we developed a supramolecular nanocomplex based on phenolic‐modified SS for systemic delivery of antioxidant enzymes across the BBB, enabling intracellular release and therapeutic activity within brain cells. The conjugation of phenolic compounds to SS achieved three goals: (1) effective protein complexation, (2) modulation of the pI of SS, and (3) enhanced anti‐inflammatory effect.

Unmodified SS is a highly hydrophilic biopolymer with intrinsic antifouling properties, which limits its direct interaction with proteins [43]. In contrast, phenolic compounds—such as catechol and pyrogallol—can form dynamic supramolecular interactions with proteins via multiple non‐covalent forces, including hydrogen bonding, π–π stacking, and hydrophobic interactions [44, 45]. Following conjugation of dopamine or gallic acid to SS, the resulting phenolic SS (SSDA and SSGA) formed stable nanocomplexes with three model proteins possessing diverse pI values under supramolecular interactions and extrusion (Figure 3A–D). CAT was chosen as a model enzymatic protein drug because it uniquely and efficiently breaks down hydrogen peroxide, directly targeting a key source of oxidative inflammation. Unlike consumable small‐molecule antioxidants, its catalytic action prevents depletion and redox imbalance. As a protein whose function depends on structural integrity and intracellular delivery, CAT serves as an ideal candidate to test our system's protective and delivery capabilities. Using CAT as a representative therapeutic enzyme, we observed significantly enhanced binding affinity between CAT and SSDA/SSGA compared to unmodified SS, as confirmed by ITC (Figure 3F–H). Both SSDA and SSGA facilitated cytosolic delivery of CAT with comparable efficiency (Figure 4C). This result is consistent with prior observations that both catechol and pyrogallol‐modified proteins promote cellular internalization [46], though different delivery efficiency of the two phenolic motif‐modified delivery systems was also reported [47].

Second, phenolic conjugation differentially modulated the pI of SS, thereby enabling selective charge‐reversal behavior under acidic conditions. SS is an acidic protein with a pI of approximately 3.9. Upon DA conjugation, carboxyl groups on SS were partially substituted by amide bonds, leading to an increased NH_2_/COOH ratio and a corresponding rise in pI. Conversely, conjugation with GA consumed partial amino groups, thereby decreasing the NH_2_/COOH ratio. As a result, SSDA exhibited an elevated pI of 4.4, whereas SSGA showed a reduced pI of 3.5 (Figure S9). The modest pI increase observed in SSDA likely reflects the partial retention of negative charges on the introduced catechol groups, consistent with prior findings [48]. Nonetheless, this upward shift was sufficient to enable pH‐responsive charge reversal: under acidic lysosomal conditions, the zeta potential of DC shifted from negative to positive, promoting lysosomal escape (Figure 3M). In contrast, GC, lacking this charge‐switching capability, remained sequestered within lysosomes (Figure 4D–F). This charge‐reversal strategy echoes our previous work [49], where the amino group in chitosan was crosslinked to SS to increase pI value, enabling mild acid‐triggered surface charge inversion.

Third, an additional advantage of phenolic SS lies in its enhanced anti‐inflammatory properties. SS itself is a natural biopolymer known for its mild intrinsic anti‐inflammatory activity [34]. The two phenolic compounds, dopamine [50] and gallic acid [51], have also been reported to be anti‐inflammatory. Upon conjugation, the resulting phenolic SS retained and, in some cases, amplified these effects. In particular, SSDA exhibited greater anti‐inflammatory activity than both SSGA and unmodified SS, as evidenced by reduced expression of inflammatory markers and proinflammatory cytokines in LPS‐stimulated macrophages (Figure 2F–H). Coupled with the inherent anti‐inflammatory effect of SSDA and enhanced lysosome escape capacity of the DC nanocomplex, SSDA provided a dual benefit—delivering catalase efficiently into the cytosol and simultaneously attenuating neuroinflammatory responses. As a result, DC significantly outperformed GC in both the H_2_O_2_‐induced oxidative stress model and the LPS‐induced inflammation model (Figure 4).

Unlike RGD or cRGD (cyclic Arg‐Gly‐Asp) peptides, iRGD is a 9‐amino acid cyclic peptide that incorporates an RGD motif but functions through a distinct mechanism. Initially, iRGD targets integrins expressed on vascular endothelial cells, followed by proteolytic cleavage that exposes a C‐terminal CendR motif. This fragment subsequently binds to neuropilin‐1 (NRP1), triggering active transcytosis across the endothelium [42, 52]. Notably, iRGD‐mediated transcytosis is a bulk transport process; once initiated, it enables nonspecific “bystander” uptake of co‐administered compounds, including nanoparticles, into underlying tissues [53]. Previous studies have demonstrated that co‐administration of iRGD significantly enhances nanoparticle accumulation in tumors via this bystander effect [41, 54, 55]. In this study, simple co‐formulation of iRGD with nanocomplexes improved BBB penetration both in vitro and in vivo in the context of AD (Figure 5; Figure S18), consistent with previous findings that iRGD‐equipped nanoparticles accumulate more effectively in the brain than iRGD‐free controls, even in non‐tumor‐bearing models [56].

Interestingly, in addition to its anti‐inflammatory and antioxidant effects, iDC treatment markedly reduced Aβ plaque deposition in the brains of APP/PS1 mice (Figure 7D,H). The result is in agreement with prior reports that anti‐oxidant and anti‐inflammatory peptide mimetic [57] and nanozymes [23, 58] reduced Aβ plaque deposition in AD mouse models. While this observation suggests a potential role for antioxidant enzyme delivery in modulating amyloid pathology, the underlying mechanisms by which iDC attenuates Aβ accumulation remain to be elucidated. Possible contributors may include indirect effects via suppression of neuroinflammation or modulation of oxidative stress pathways known to influence amyloid metabolism.

While this study demonstrates the potential of a supramolecular nanocomplex for protein delivery in AD, several limitations warrant consideration. First, although iRGD facilitated BBB crossing in murine models, the mechanism of transcytosis remains indirect. Use of CendR peptide directly targeting NRP1 may improve BBB penetration efficiency. Second, the use of CAT as a model antioxidant enzyme, while mechanistically informative, may not fully capture the complexity of human AD pathology, which involves multiple interacting pathways beyond oxidative stress and inflammation. Additionally, long‐term biocompatibility, potential immunogenicity of the phenolic SS after repeated dosing were not evaluated.

## 4 Conclusions

In conclusion, our supramolecular nanocomplex platform provides a modular, biocompatible, and functionally integrated solution for the intracellular delivery of therapeutic proteins to the brain. By combining BBB permeability, lysosomal escape, and intrinsic anti‐inflammatory activity, this strategy offers a promising blueprint for next‐generation nanomedicine design targeting other neurodegenerative and inflammatory brain disorders.

## 5 Experimental Section

### 5.1 Materials and Animals

Silk sericin (MWCO 30 000–40 000 Da) was purchased from Xintiansi Biotechnology Co., Ltd (Huzhou, China). Dopamine HCl, 3,4,5‐Trihydroxybenzoic acid, 1‐ethyl‐3‐(3‐(dimethylamino)propyl) carbodiimide hydrochloride (EDC·HCl), N‐Hydroxysuccinimide (NHS), and 1,1‐Diphenyl‐2‐picrylhydrazyl (DPPH) were supplied by Energy Chemical Co., Ltd (Shanghai, China). Fluorescein isothiocyanate (FITC) and 2‐Morpholinoethanesulphonic acid (MES) were purchased from Macklin Biochemical Co., Ltd (Shanghai, China). Cy5.5‐NHS ester was purchased from Aladdin Biochemical Technology Co., Ltd (Shanghai, China). DCFH‐DA was purchased from Acmec Biochemical Co., Ltd (Shanghai, China). Catalase (CAT, 2000–5000 units/mg protein) was purchased from Sigma–Aldrich, Inc. LPS, rabbit anti‐Arg1 antibody (AF1381), Lyso‐Tacker red (C1046), and Catalase Assay Kit were purchased from Beyotime Biotech. Inc. iRGD was purchased from GL Biochem Co., Ltd (Shanghai, China). Folin's Phenol Regent was supplied from Sinopharm Chemical Reagent Co., Ltd (Beijing, China). Rabbit anti‐iNOS antibody (#13120) was purchased from Cell Signaling Technology. Inc. TNF‐α, IL‐6, and IL‐10 were supplied from Elabscience Biotechnology Co., Ltd (Wuhan, China). Anti‐CD86 antibody (83213‐1‐RR), anti‐CD206 antibody (81525‐1‐RR), and anti‐integrin αv antibody (27096‐1‐AP) were supplied from Proteintech Group, Inc. (Wuhan, China). Anti‐DNA/RNA Damage antibody [15A3] (ab62623), and Anti‐Neuropilin 1 antibody (ab81321) were supplied from Abcam Co., Ltd. Anti‐Aβ antibody (6E10, SIG‐39320) was purchased from BioLegend, Inc. Mouse/Rat Neuropilin‐1 blocking antibody (AF566) was purchased from R&D systems, Inc. C57BL/6 female mice aged 8 weeks were obtained from Bestest Biotechnology Co., Ltd (Zhuhai, China). APP/PS1 female mice aged 9 months were obtained from Huachuang Sino Pharmatech Co., Ltd (Jiangsu, China). All animal protocols were approved by the Institutional Animal Care and Use Committee and used following the provisions of Animal Experimentation Committee of South China Agricultural University (approval number: 2023b189).

### 5.2 Synthesis and Characterization of SSDA/SSGA

SSDA and SSGA, two phenolic SS, were synthesized through EDC/NHS chemistry. For SSDA synthesis, SS (200 mg) was dissolved in 20 mL MES buffer (pH 6.0, 0.1 mm) containing EDC (77 mg) and NHS (57 mg). The solution was magnetically stirred for 1 h. Then, dopamine hydrochloride (DA) (300 mg) was added to the solution in an atmosphere of nitrogen, and the reaction was allowed to last for 24 h at room temperature. Then, the solution was centrifuged at 7000 rpm for 10 min to remove aggregates before lyophilizing into SSDA powder. For SSGA synthesis, gallic acid (GA) solution (272 mg, 1.6 mmol) was activated by EDC (250 mg, 1.6 mmol) and NHS (185.6 mg, 1.6 mmol) in MES buffer (pH 6.0, 0.1 mm) for 0.5 h. Then, SS (200 mg, 0.4 mmol) was added to the solution and reacted for 24 h. Lyophilized SSGA powder was used for further experiments. The content of polyphenols in SSDA or SSGA was determined by Folin‐Ciocalteu assay. The structures of SSDA or SSGA were determined by FT‐IR and circular dichroism spectra (CD). The antioxidant activities of SSDA and SSGA were determined by DPPH radical scavenging assay.

### 5.3 Synthesis of FITC‐Labeled CAT

CAT powder (30 mg) was dissolved in 20 mL PBS, and pH was adjusted to 8.5 with 1 m NaHCO_3_. FITC (6 mg) dissolved in DMSO was added to the solution and reacted for 24 h in the dark at room temperature. FITC‐labeled CAT (^FITC^CAT) was obtained following dialyzing (MWCO 3500 Da) and lyophilization.

### 5.4 Preparation and Characterization of SSDA/SSGA‐Based Protein Delivery Systems

First, BSA, lysosome, horseradish peroxidase, and CAT were selected as protein candidates based on their isoelectric point (pI) or biological activities. Then, proteins and SSDA/SSGA were dissolved in PBS (pH 7.4), and the mixture of protein and SSDA/SSGA was rigorously stirred for 10 min at a weight ratio of 1:20. The mixture was transferred to a mini‐extruder equipped with a 200 nm membrane and extruded 15 times to obtain protein‐loaded nanoparticles. The CAT‐loaded nanoparticles complexed by SSDA or SSGA were named DC or GC, respectively. DC or GC was subjected to size‐exclusion chromatography using a Sephadex G‐200 column, with PBS (pH 7.4) as the elution buffer. The amount of unencapsulated CAT was quantified by measuring absorbance at 405 nm, corresponding to the Soret band. The encapsulation efficiency (EE) was calculated according to the following equation:

(1) $$\mathrm{EE}=(M_{\mathrm{total}\mathrm{CAT}}-M_{\mathrm{unloaded}\mathrm{CAT}})/M_{\mathrm{total}\mathrm{CAT}}\times 100\%$$

where M_total CAT_ represents the total mass of CAT used, and M_unloaded CAT_ denotes the mass of unloaded CAT. For CAT release study, DC or GC suspensions were incubated in PBS at pH 7.4 and pH 5.0 under constant agitation (200 rpm) at 37°C. At predetermined time intervals (1, 3, 6, and 12 h), aliquots were withdrawn and immediately applied to a Sephadex G‐200 column to separate the released CAT from the encapsulated fraction, allowing quantification of the cumulative release over time. The particle size and zeta potential under different pH values of DC and GC were examined by Zetasizer Nano ZS90 (Malvern Panalytical). The morphological examinations were performed by transmission electron microscopy (TEM). Fluorescence quenching assay was recorded by fluorescence spectroscopy. Briefly, to a solution of ^FITC^CAT, SSDA, or SSGA was added in a series of ratios. The process was recorded with an excitation wavelength of 488 nm and emission wavelength of 500–600/650 nm. To study the mechanism of nanocomplex formation, NaCl (100 mm), urea (400 mm), and Tween 20 (0.2% (v/v)) was added to ^FITC^CAT, ^FITC^DC, or ^FITC^GC. Fluorescence intensity of FITC was recorded following incubation for 1 h. The interactions between SSDA/SSGA and CAT were measured by isothermal titration calorimetry (ITC, MicroCal PEAQ‐ITC, Malvern). Briefly, the ITC titration was conducted by titrating 19 aliquots of sericin derivatives (250 µm, PBS) to CAT (10 µm, PBS) at T = 25.0°C, and the heat evolution was recorded. CAT enzymatic stability test was performed by incubating FC, DC, and GC in the presence of proteinase K (0.025 mg/mL) at 37°C for 4 h. CAT activity in DC and GC was determined by catalase assay kit according to the manufacturer's protocol (S0051, Beyotime).

### 5.5 Cell Culture and In Vitro Cytotoxicity

N2a, BV2, and bEnd.3 cells, provided by Cell Bank/Stem Cell Bank of the Chinese Academy of Sciences, were cultured in DMEM medium, supplemented with 10% fetal bovine serum and 1% penicillin/streptomycin. In vitro cytotoxicity was assessed using the CCK‐8 kit (Beyotime, Shanghai, China). Cells were seeded into 96‐well plates at a density of 1×10^4^ cells per well and incubated with DC and GC for 24 h. At the designated time points, the medium was replaced with a 10% (v/v) CCK‐8 solution. After incubation at 37°C for 1 h, the absorbance of the solution was measured at 450 nm using a microplate reader (Multiskan FC, Thermo, USA).

### 5.6 In Vitro Cellular Uptake and Co‐Localization Toward DC and GC

N2a and BV2 cells were seeded in confocal dishes (1 × 10^5^) and incubated for 24 h. The cells were incubated with free ^FITC^CAT, ^FITC^DC, and ^FITC^DC (10 µg/mL ^FITC^CAT) for different times. For confocal laser scanning microscopy (CLSM) observation of cellular uptake, the cells following incubation were rinsed with PBS and stained with Hoechst33342 before CLSM observation. For intracellular co‐localization, the cells were incubated with free ^FITC^CAT, ^FITC^DC, and ^FITC^DC for a specified time. After incubation, the cells were rinsed and loaded with LysoTracker Red and Hoechst 33342 before CLSM observation. The co‐localization was analyzed with ImageJ.

### 5.7 H_2_O_2_‐Induced Oxidative Damage Model and Intracellular ROS Determination

H_2_O_2_‐induced cytotoxicity: H_2_O_2_‐induced cytotoxicity was determined by CCK8 assay. N2a cells (1× 10^4^) were seeded in 96‐well plates for 24 h. DC and GC were incubated for 24 h before H_2_O_2_ (200 µm) incubation for another 12 h. At the end of incubation, the cell culture medium was aspirated and supplemented with fresh medium, followed by incubation with 10 µL of CCK8 at 37°C. The absorbance of solutions was monitored at 450 nm on a microplate reader. ROS detection: N2a cells (1 × 10^5^) were seeded in 24‐well plates for 24 h and incubated with DC and GC (10 µg/mL CAT) for 24 h. Then, the media were replaced by H_2_O_2_ (200 µm). Free CAT (FC) was treated as control. Following 12 h incubation, cells were loaded with ROS probe DCFH‐DA (10 µm) in medium without FBS for 20 min at 37°C. Then, the cells were harvested for flow cytometry analysis. Similarly, N2a cells cultured in a confocal dish were treated likewise and imaged by CLSM.

### 5.8 LPS‐Induced Inflammatory Model and Microglial Polarization

Inflammatory model was established by incubating BV2 cells with LPS (1 µg/mL) for 24 h. The cells were incubated with SS, SSDA, and SSGA (200 µg/mL) for 24 h, respectively. Then, the cells were harvested for Western blot analysis, and supernatants were collected for cytokine analysis. Meanwhile, in the presence of LPS, FC, DC, and GC with a CAT concentration of 10 µg/mL, were added and incubated for 24 h. To visualize the expression of polarization markers of BV2, the treated cells were fixed in 4% paraformaldehyde for 15 min, permeabilized with 0.1% Triton X‐100 for 5 min, and blocked with 1% BSA for 30 min. Following washing with PBS, the cells were incubated overnight at 4°C with the primary antibody against CD86 (Protentech, #83213‐1‐RR) and CD206 (Protentech, #81525‐1‐RR) at 1: 200. Then, the cells were incubated with Alexa Fluor 488‐conjugated secondary antibody (ThermoFisher, #A‐11008) at 1:500 at room temperature and stained with DAPI before CLSM observation. Furthermore, in the presence of LPS, BV2 cells were incubated with DC, SSDA‐BSA (DB), GC, and SSGA‐BSA (GB) at a CAT concentration of 10 µg/mL for 24 h before immunofluorescence imaging. Before cell fixation, the supernatants of BV2 were collected and centrifuged (1100 g, 20 min) for determination of cytokines measured by ELISA.

### 5.9 In Vitro BBB Penetration

bEnd.3 cells (1 × 10^5^) were seeded in the upper chambers (24 wells, pore size 0.4 µm). The culture medium was replaced every other day. bEnd.3 cell monolayer in a transwell system was established by culturing for 7 days as the in vitro BBB model. The TEER of the bEnd.3 monolayers was detected by Millicell ERS 3.0 (Millipore, USA). ^FITC^DC, ^FITC^GC, ^FITC^iDC, and ^FITC^iGC were added in the upper chambers with an iRGD concentration of 50 µg/mL. After incubation for 4, 8, 12, and 24 h, the liquid in the down chambers was harvested for fluorescence assay (ex: 488 nm, em: 515 nm) in a microplate reader. Meanwhile, N2a cells were seeded in the lower chamber of the transwell system. ^FITC^DC, ^FITC^GC, ^FITC^iDC, and ^FITC^iGC were added in the upper chambers and incubated for 24 h. Then, the cells in the lower chamber were fixed for imaging by CLSM.

### 5.10 In Vivo Distribution of iDC and iGC

Cy5.5‐labeled DC/GC (^Cy5.5^DC/GC) were intended for in vivo biodistribution. To prepare ^Cy5.5^DC/GC, the pH of DC/GC in PBS was adjusted with 1 m NaHCO_3_ to pH 8.0. Cy5.5 dissolved in DMSO was added to the solution and reacted for 24 h in the dark at room temperature. ^Cy5.5^DC/GC were obtained following dialyzing against PBS (pH 7.4). The resulting solutions were stored at 4°C for use. Female C57BL/6 mice were randomly divided into 4 groups (n = 3). ^Cy5.5^DC/GC or ^Cy5.5^iDC/iGC were given once via tail vein at a dose of iRGD (10 mg/kg) and DC/GC (100 mg/kg). At 1, 3, 6, 9, and 12 h post‐administration, mice were anesthetized and imaged via in vivo imaging system spectrum (PerkinElmer, USA). Then, mice were sacrificed to obtain major organs, including the brain, for ex vivo fluorescence imaging. After imaging, brains were fixed with 4% paraformaldehyde, embedded, and frozen‐sectioned. Then, the slices were stained with DAPI for fluorescence observation. Meanwhile, female APP/PS1 mice were randomly divided into 3 groups (n = 3), ^Cy5.5^DC, ^Cy5.5^iDC, and ^Cy5.5^iDC + anti‐NRP1 blocking antibody (αNRP1) were intravenously injected to the mice. αNRP1 of 30 µg was administrated to the mice 15 min before nanocomplex injection. To observe the expression of α_v_β_3_ and NRP1 in APP/PS1 mice, the mice were euthanized, and brains were collected for immunofluorescence imaging following fixation, embedment, frozen‐section, and staining.

### 5.11 Behavior Test

APP/PS1 mice (9 months old) were randomly divided into 4 groups (n = 5), and wild‐type (WT) C57BL/6 mice (n = 5) were the normal control group. Mice in the four AD groups were intravenously injected with PBS, iFC, iDC, and iGC. WT mice were injected with PBS. CAT dose was 10 mg/kg. The mice received one injection every week, and the treatment lasted for about two months. For Morris water maze experiment, the maze was made up with a circular pool (diameter: 120 cm; height: 50 cm) and an escape platform (diameter: 9 cm). The tank was divided into four equal quadrants. During the first five days of training, mice were placed in one quadrant randomly and allowed to swim freely until they found the escape platform within 60 s. If mice failed to find the platform, they were guided and kept on the platform for 15 s. The course was repeated three times daily. Swimming paths and escape latencies (the time until mice reached the escape platform) of mice were recorded by a video‐tracking system. With the escape platform removed on the sixth day, mice were released in the tank for 60 s. Spatial memory indexes, including the time spent in targeted quadrant and the number of crossing escape platform location, were recorded. Nesting test: Before the test, mice were transferred to individual cages for environmental adaptation. Then, three pieces of tissues (15 cm × 17 cm) were placed in each cage for mice to build nests. After 24 h, the nest was photographed, and the scoring was performed in a blinded manner. The point scale is as follows: 1, the tissues were not touched; 2, less than 50% of the tissues were used for nest construction; 3, the tissues were concentrated while some of them were scattered; 4, a significant nest can be identified; 5, almost all of the materials were used for nest construction.

### 5.12 Histological Analysis

After the treatment, brain and major organs of the mice were collected and fixed in 4% paraformaldehyde overnight and embedded in paraffin wax. Sections of brain were stained with immunochemistry and immunofluorescence to characterize the oxidative damage, inflammation, microgliosis, Aβ deposition, and neuron protection. Oxidative damage was marked by anti‐DNA/RNA Damage antibody, inflammation was marked by anti‐TNF‐α antibody, microglia were marked by anti‐Iba‐1 antibody, and Aβ deposition was marked by anti‐Aβ antibody. Neuron was marked by Nissl staining and anti‐Neun antibody. Major organs were stained with hematoxylin and eosin (H&E) to evaluate biocompatibility of the nanocomplexes. Image J software was utilized for quantification analysis.

### 5.13 Statistical Analysis

Data were reported as mean ± standard deviation (SD). All statistical comparisons and nonlinear regressions were performed in Prism 8.3 (GraphPad Software, Inc.). One‐way analysis of variance (ANOVA) with Bonferroni test or unpaired student's t‐test was used to compare data from more than two groups or two groups, respectively. *p*‐value <0.05 was considered to be statistically significant.

## Conflicts of Interest

None of the authors have a conflict of interest to disclose.

## Supporting information




**Supporting File**: advs75342‐sup‐0001‐SuppMat.docx.

## Data Availability

The data that support the findings of this study are available from the corresponding author upon reasonable request.
