# Supplementary material for: Dual Physiological Barriers Bypassed by a Silk‐Based Supramolecular Protein Delivery Platform for Neuroinflammation Mitigation in Alzheimer's Disease
Source: Adv Sci (Weinh). 2026 Apr 20;13(38):e75342. doi: 10.1002/advs.75342 (PMC13335438; doi:10.1002/advs.75342)
Supplement: Supplementary file 1 — Supporting File: advs75342‐sup‐0001‐SuppMat.docx. [file ADVS-13-e75342-s001.docx]

Supporting Information

Dual Physiological Barriers Bypassed by a Silk-Based Supramolecular Protein Delivery Platform for Neuroinflammation Mitigation in Alzheimer’s Disease

Doudou Hu*, Tiandong Li, Jian He, Yujian Jiang, Yeyuan Wang, Jingchen Sun*

D. Hu, T. Li, J. He, Y. Jiang, Y. Wang, J. Sun

Guangdong Engineering Technology Research Center of Sericulture,

College of Animal Science

South China Agricultural University

Guangzhou, Guangdong, 510642, China
E-mail: ddhu@scau.edu.cn (D.Hu); cyfz@scau.edu.cn (J.Sun)

D. Hu, T. Li, J. He
State Key Laboratory of Quality Research in Chinese Medicine

Institute of Chinese Medical Sciences

University of Macau

Taipa, Macau SAR, 999078, China


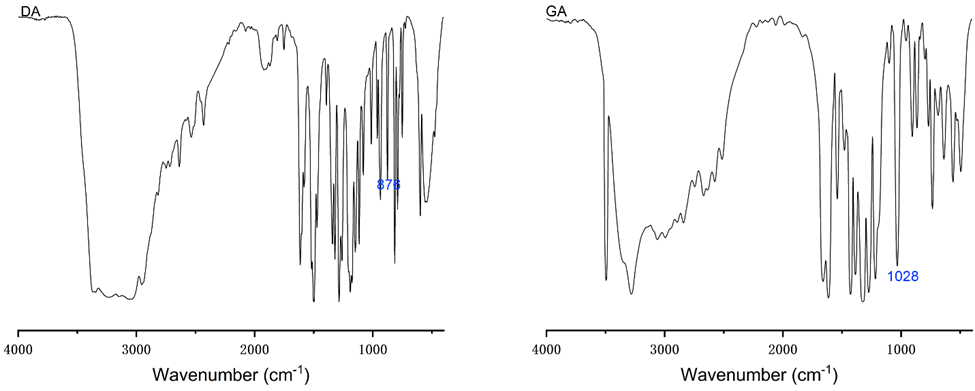


**Figure S1.** FT-IR spectra of DA and GA.


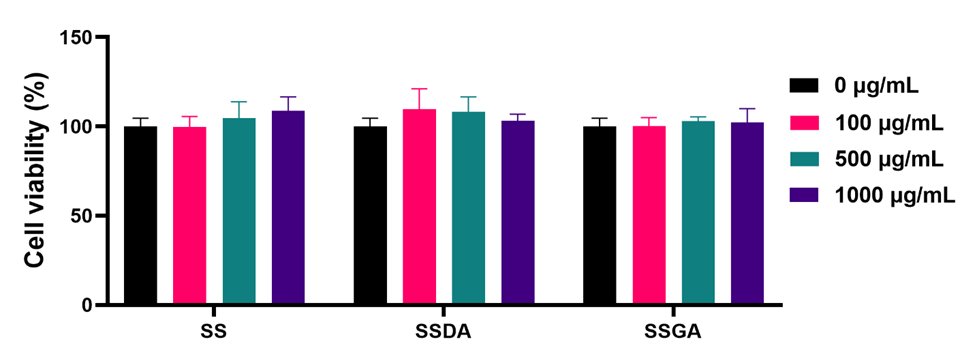


**Figure S2.** Cell viability of N2a following the incubation of SS, SSDA, and SSGA for 24 h, Data represent mean ± SD, n=5.


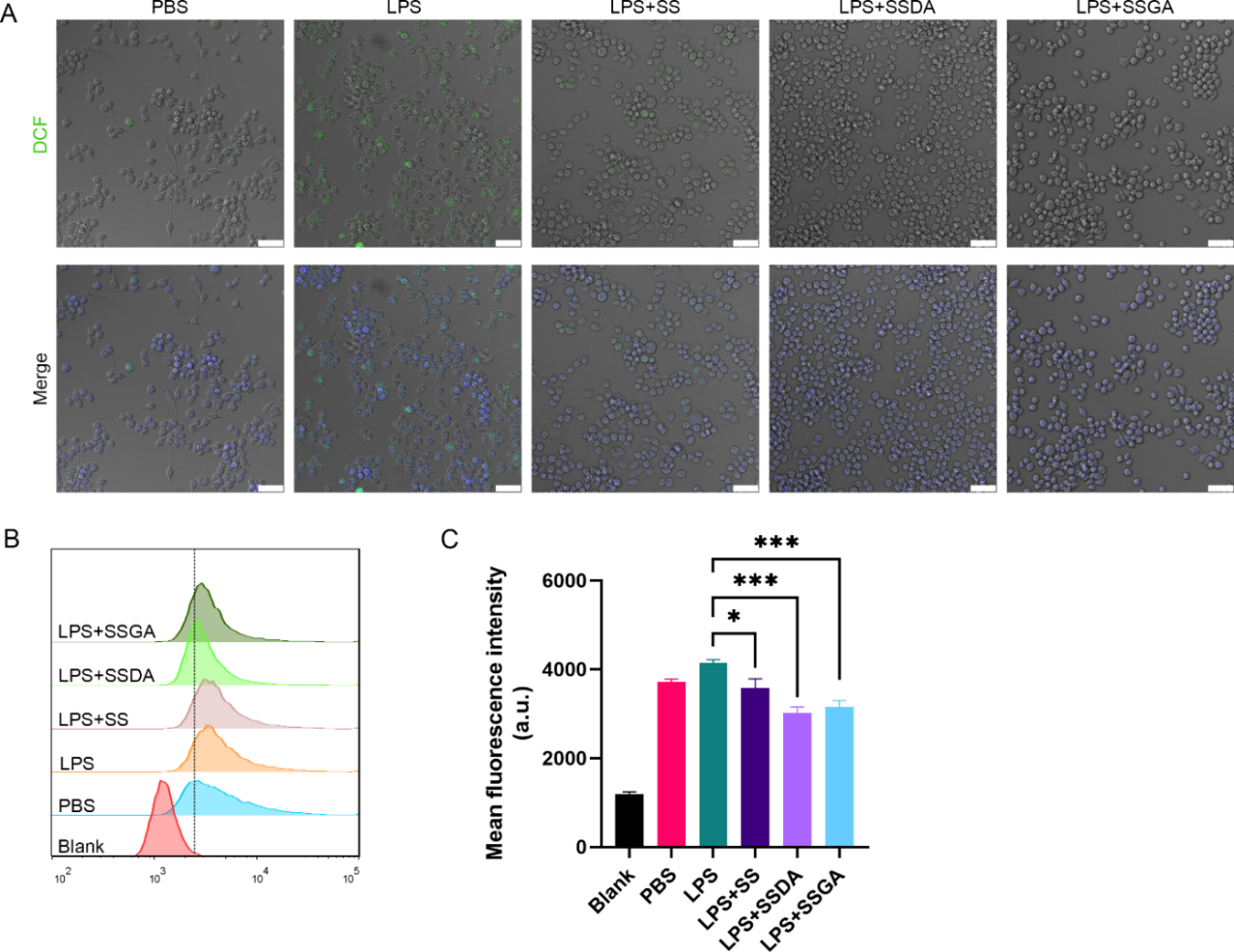


**Figure S3.** (A, B) Cellular ROS level in Raw264.7 cells with different treatments for 24 h and stained with DCFH-DA (green) determined by CLSM (A) and flow cytometry (B). Scale bar, 50 μm. (C) Mean fluorescence intensity of ROS in (B). Data represent mean ± SD, n=3; **P* < 0.05, ****P* < 0.001.
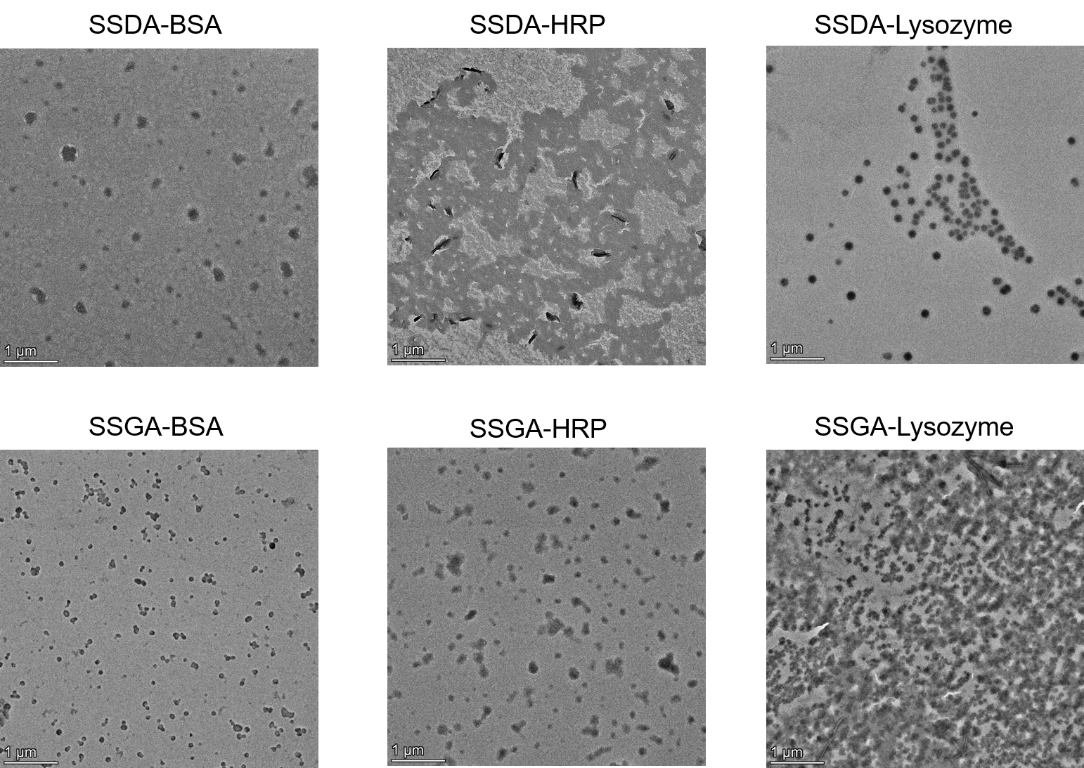

**Figure S4**. TEM images of nanocomplexes encapsulating different proteins. Scale bar, 1 μm.


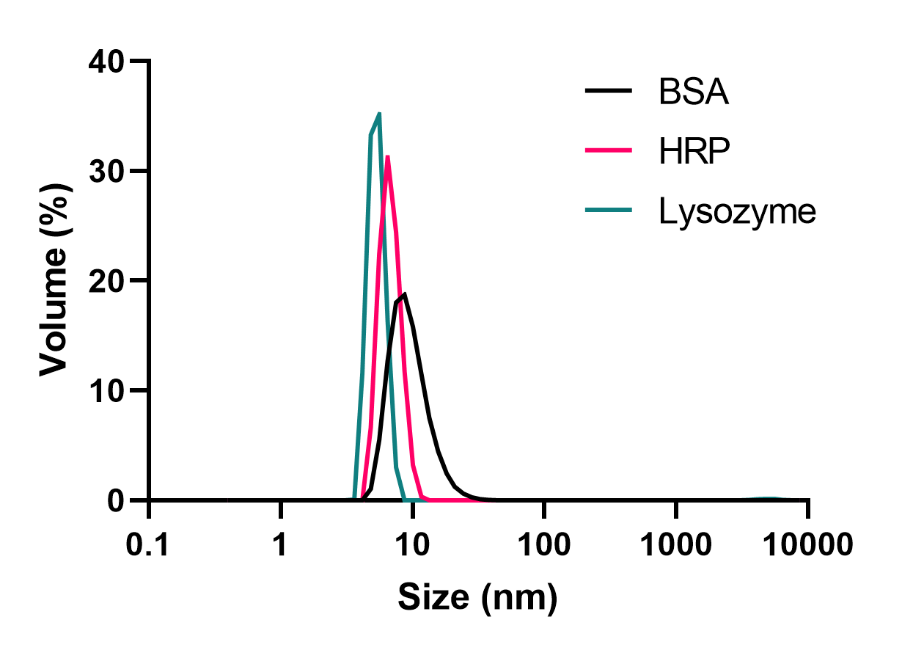


**Figure S5.** Size distribution of BSA, HRP, and lysozyme in PBS.


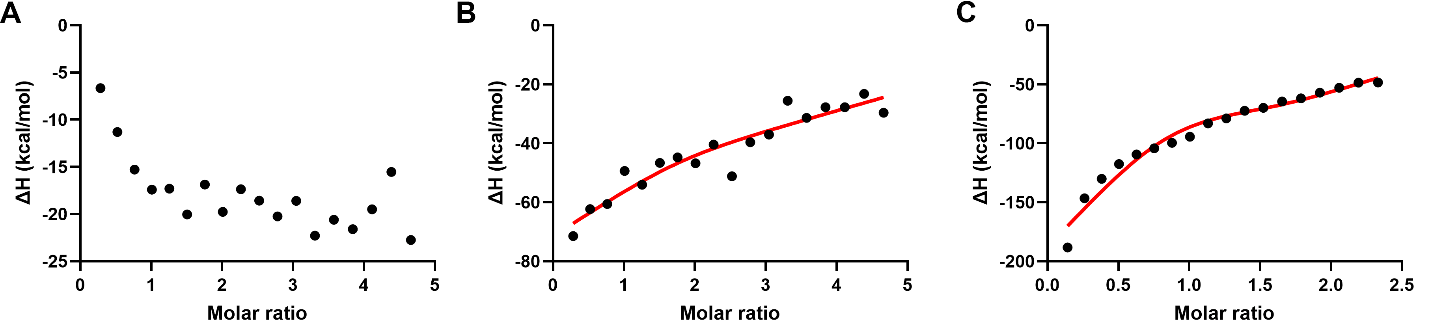


**Figure S6.** ITC raw data of (A) SS-CAT interaction, (B) SSDA-CAT interaction, and (C) SSGA-CAT interaction.


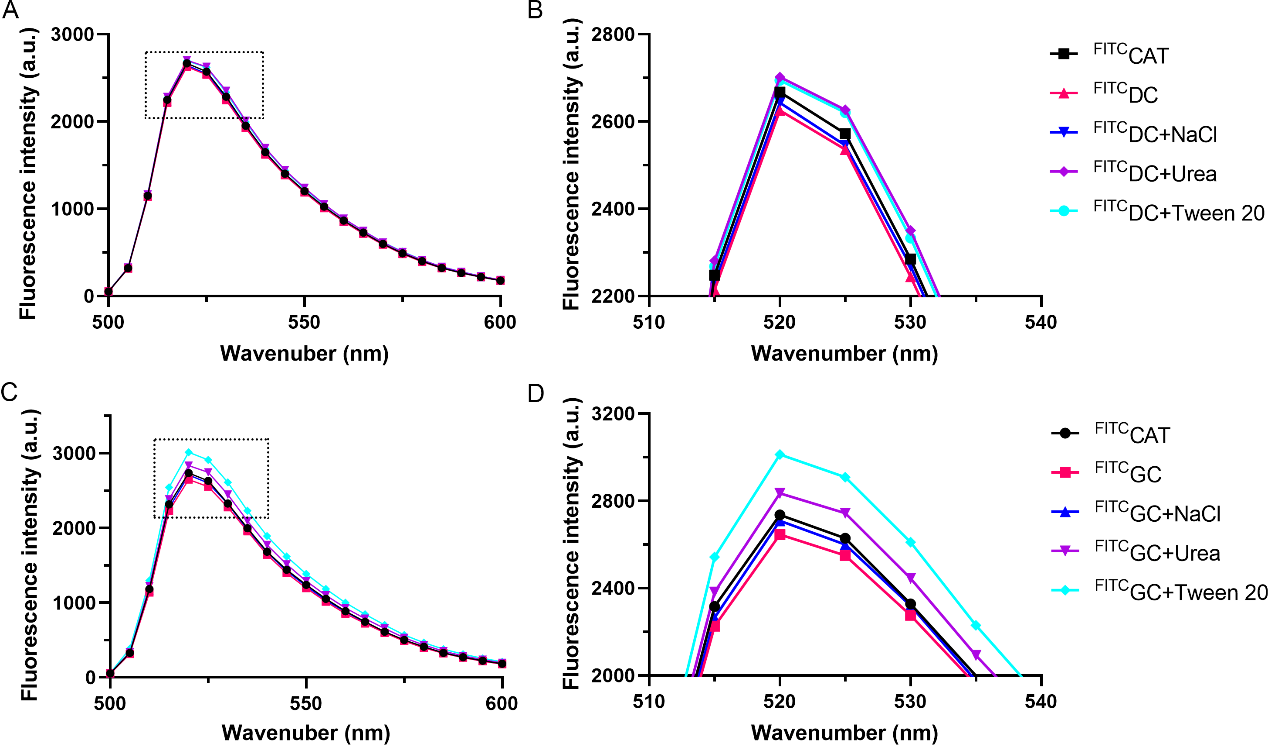


**Figure S7**. Fluorescence spectra of FITC-labeled CAT following the addition of NaCl, urea, and Tween 20.


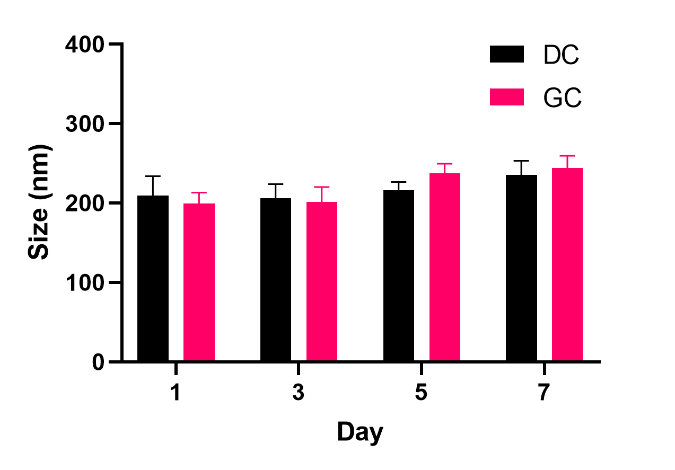


**Figure S8**. Size stability of DC and GC in PBS. Data represent mean ± SD, n=3.


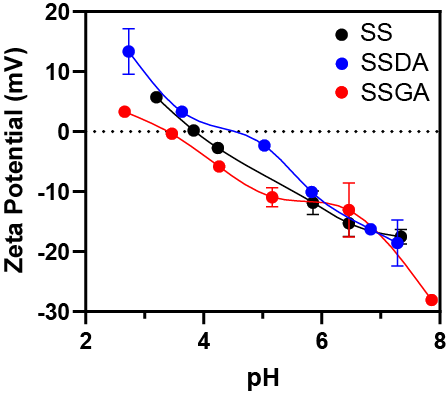


**Figure S9.** Zeta potential of SS, SSDA, and SSGA in PBS buffer with varying pH. Data represent mean ± SD, n=3.


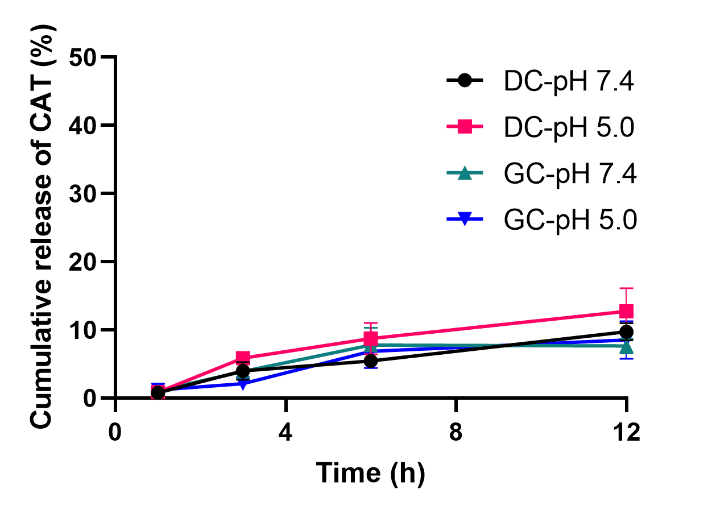


**Figure S10**. Release profile of CAT in DC and GC at pH 7.4 and pH 5.0 at 37℃. Data represent mean ± SD, n=3.


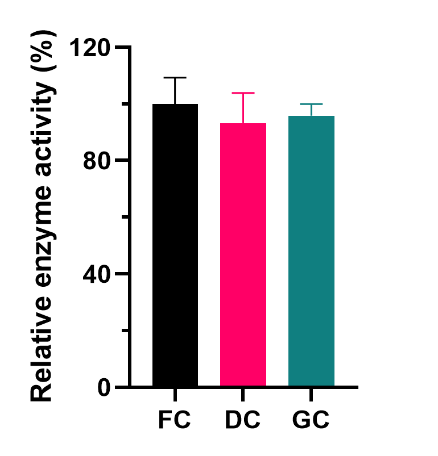


**Figure S11**.Relative CAT enzyme activity in DC and GC. Data represent mean ± SD, n=3.


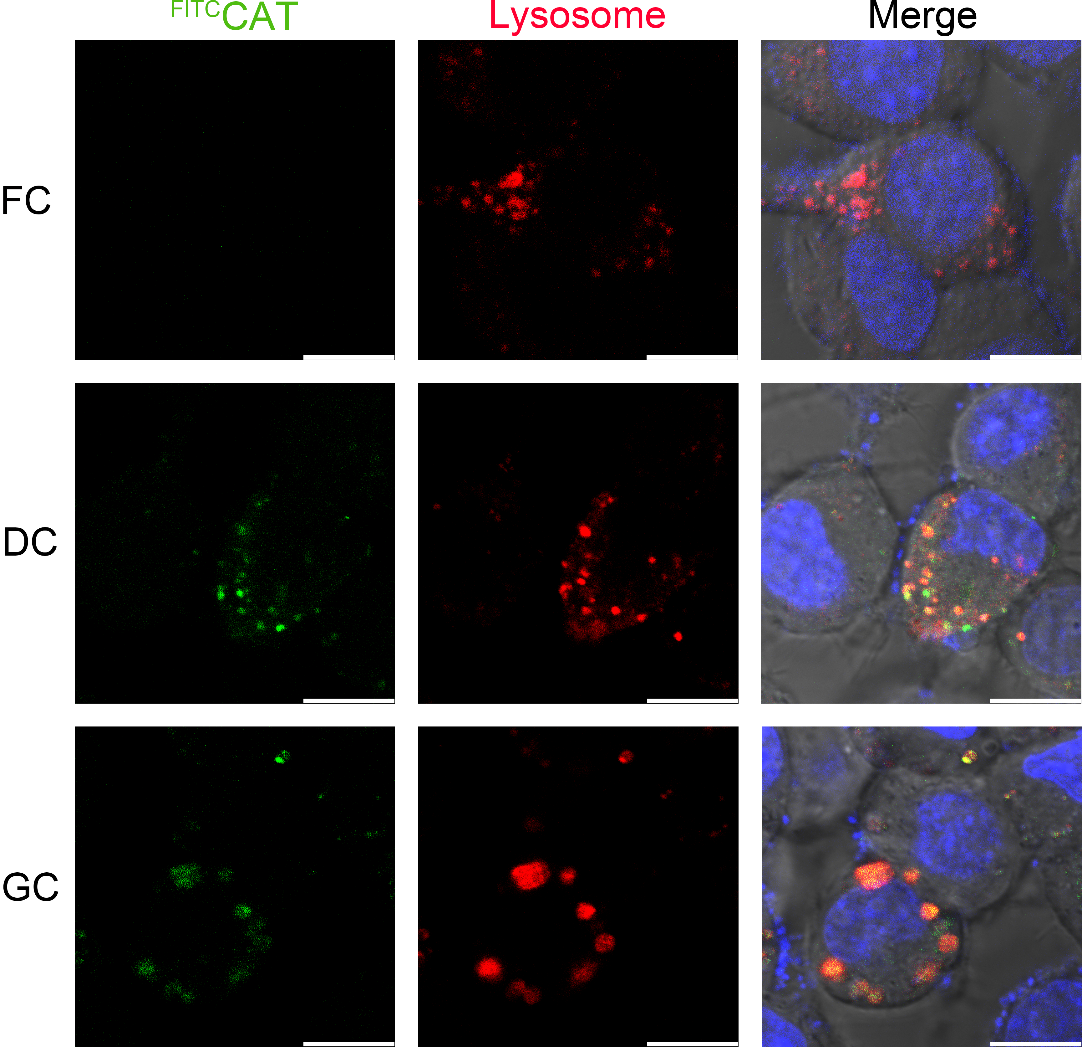


**Figure S12.** CLSM images of co-localization of FC, DC, and GC with lysosomes in N2a cells at 8 h. Scale bar, 10 μm.


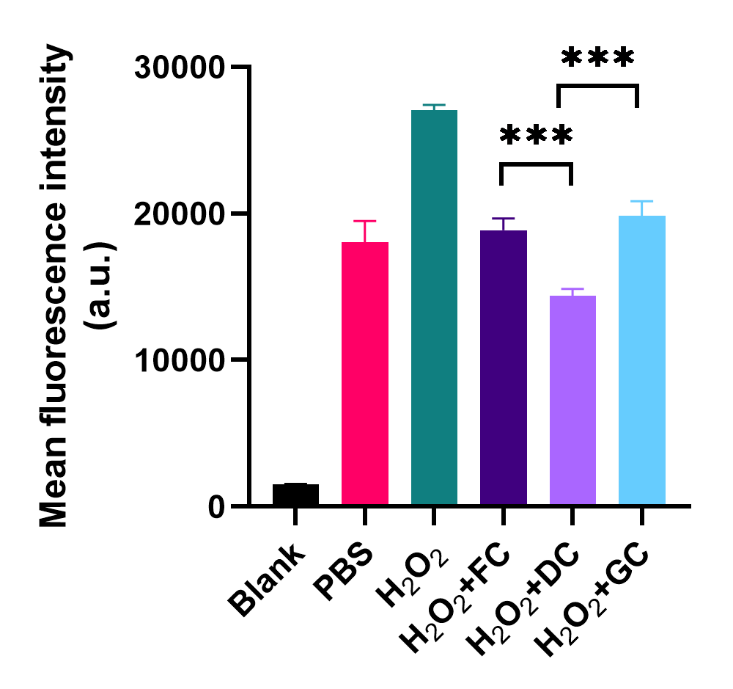


**Figure S13.** Mean fluorescence intensity of ROS in N2a cells following different treatments determined by flow cytometry. Data represent mean ± SD, n=3.


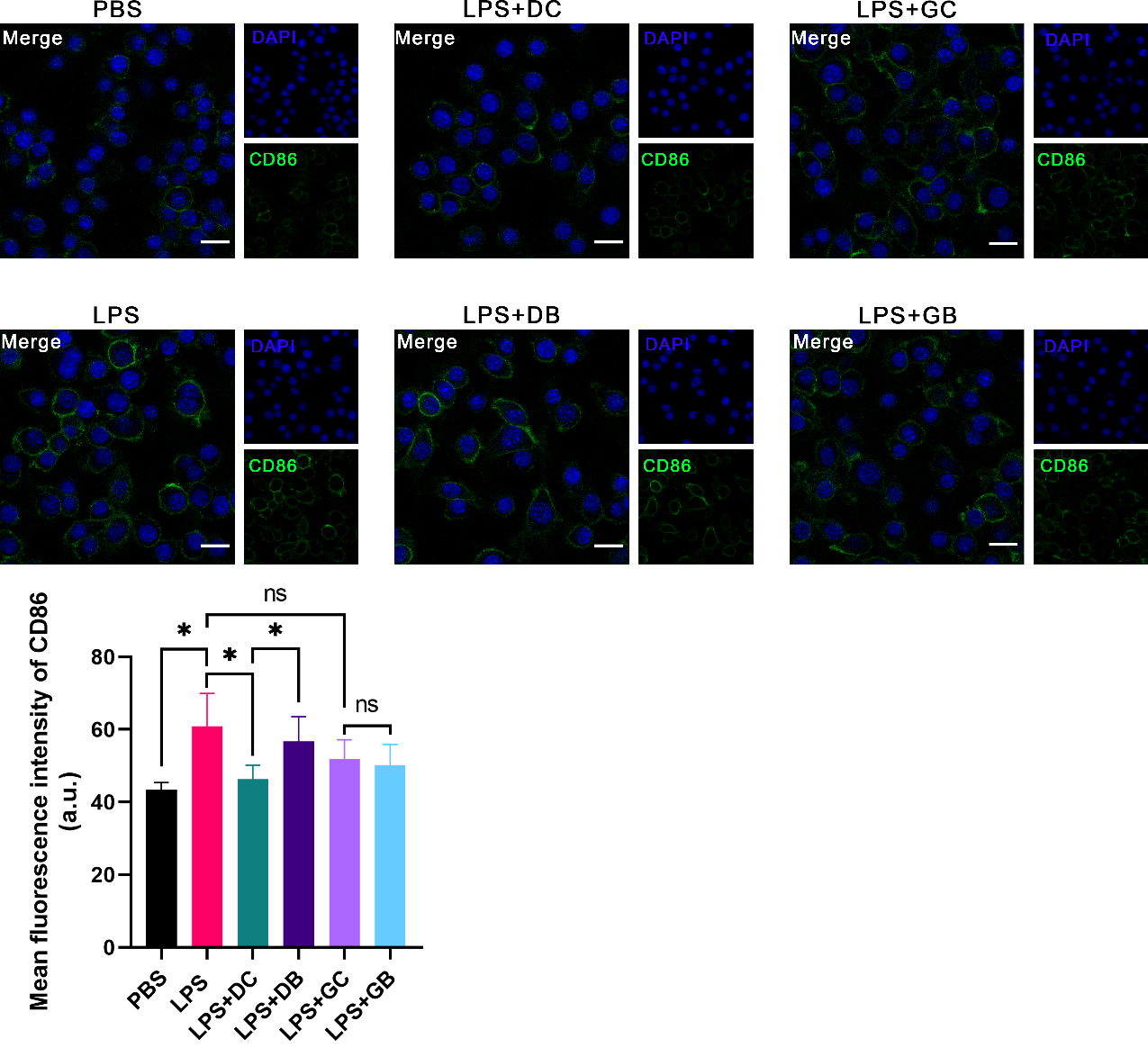


**Figure S14**. Immunofluorescence images and quantification of CD86 expression in LPS-stimulated BV2 cells following treatment for 24 h. Scale bar, 20 μm. DB: SSDA-BSA nanocomplex, GB: SSGA-BSA nanocomplex. Data represent mean ± SD, n=3.


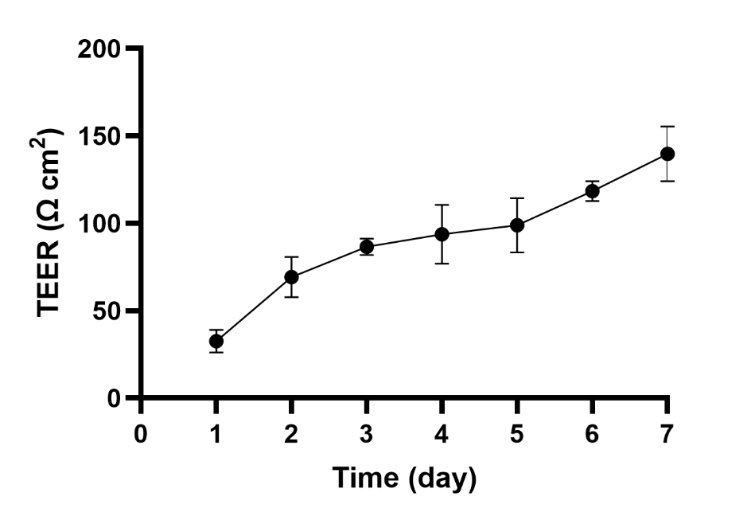


**Figure S15**. Transepithelial electrical resistance (TEER) values of bEnd.3 cells. Data represent mean ± SD, n=3.





**Figure S16.** Ex vivo distribution of Cy5.5 labeled nanocomplexes in major organs post 12 h administration.


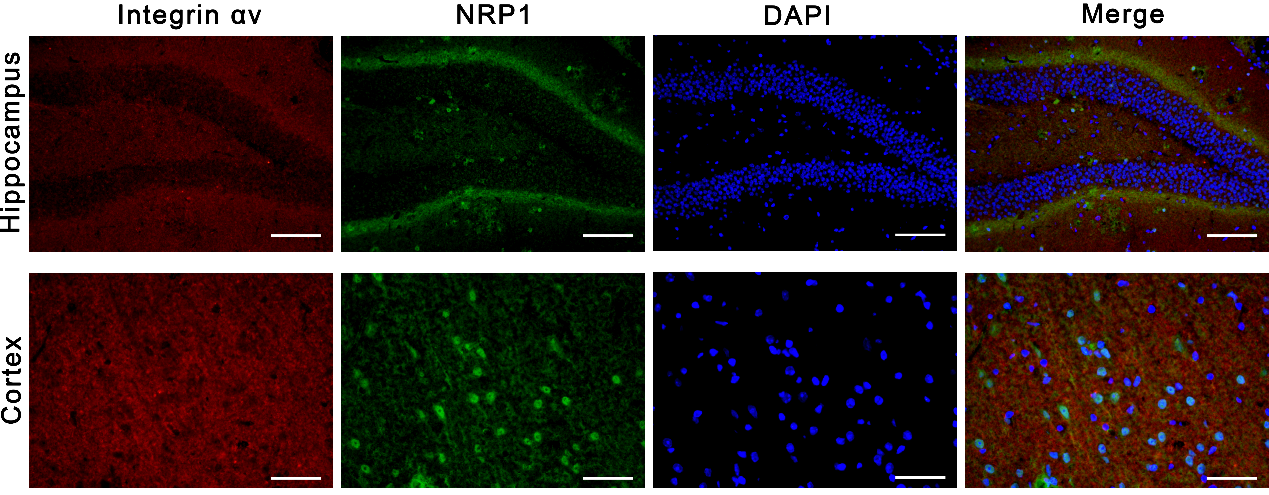
**Figure S17**. Immunofluorescence images of integrin αv and NRP1 expression in brains of APP/PS1 mice. Scale bar, 100 μm.


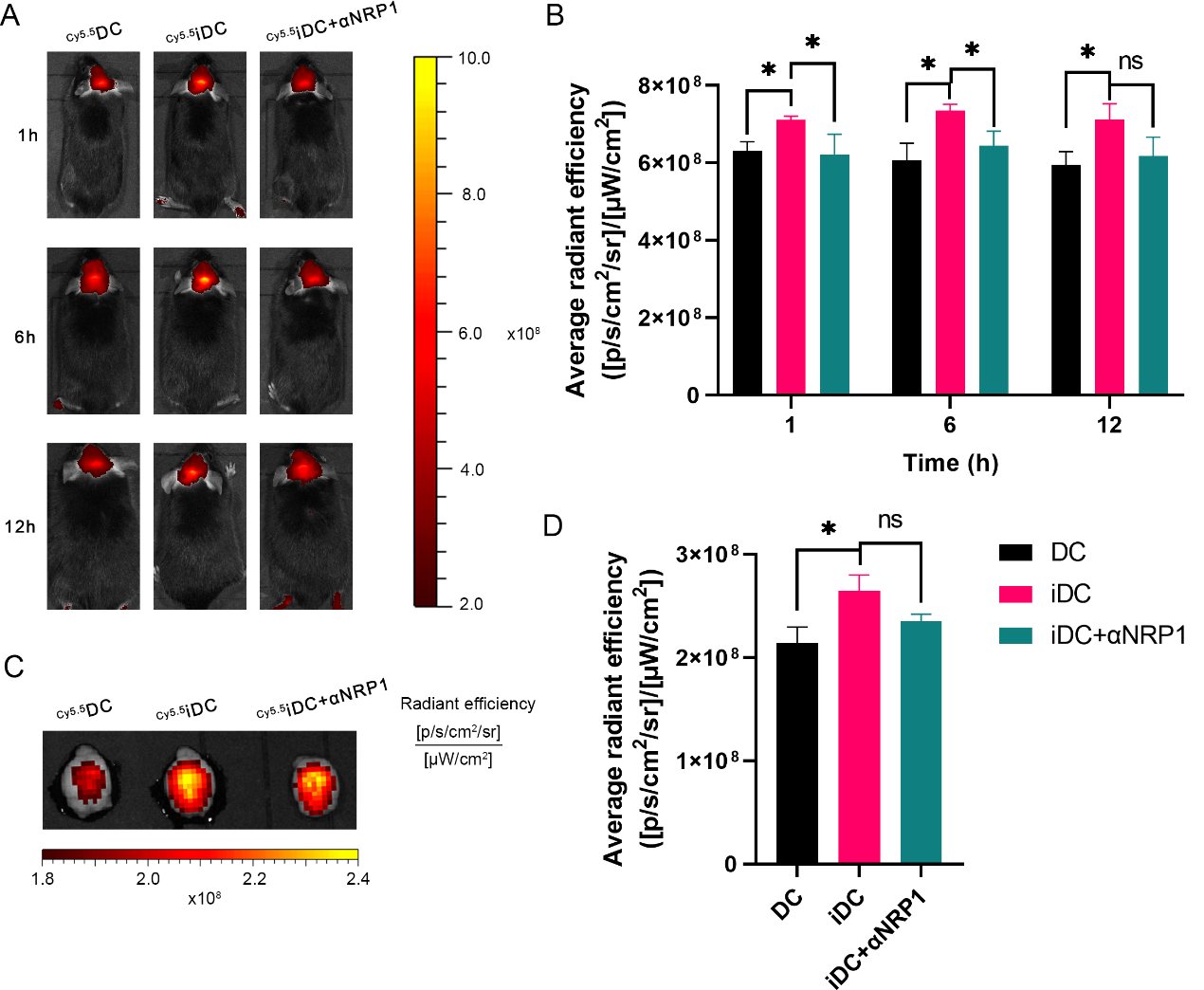


**Figure S18**. (A) In vivo fluorescence imaging of APP/PS1 mice at indicated time points following intravenous administration of Cy5.5-labeled nanocomplexes. NRP1 blocking antibody (αNRP1) was injected 15 min before nanocomplex administration. (B) Semiquantitative analysis of brain fluorescence intensity from (A). (C) Ex vivo fluorescence imaging of excised brains 12 h post-injection. (D) Quantification of brain fluorescence intensities from (C). Data represent mean ± SD (n=3); **P* < 0.05, ns, no significant.


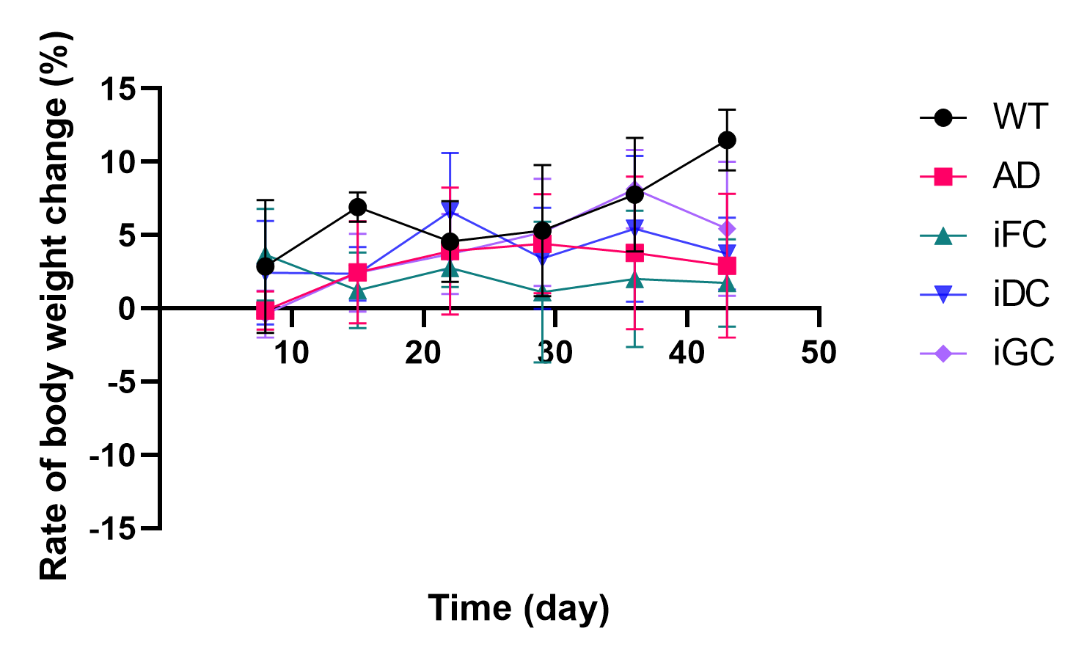


**Figure S19.** Body weight changes of WT and APP/PS1 mice during treatment. Data represent as mean ± SD, n = 5.


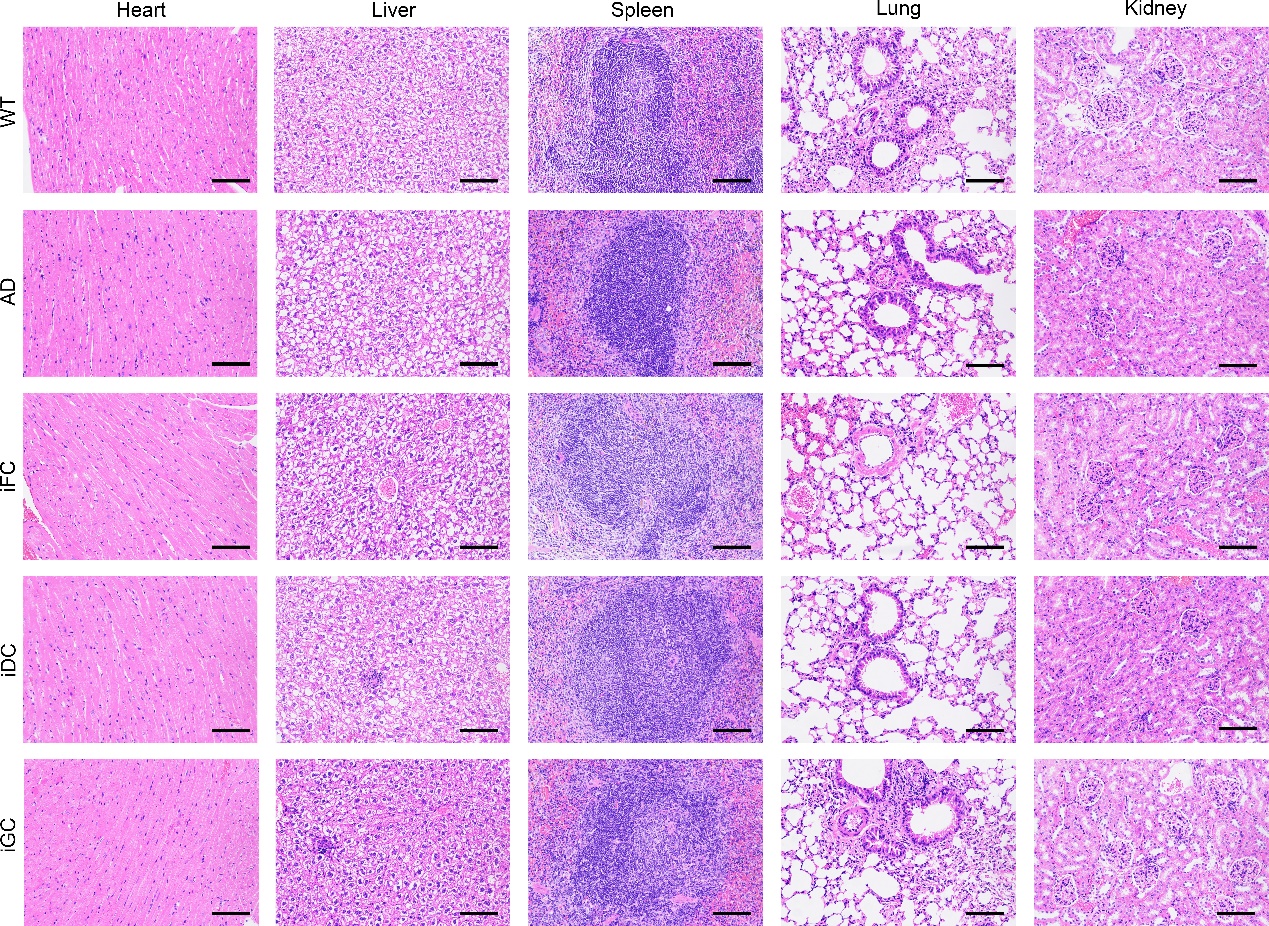
**Figure S20.** H&E sections of major organs in mice after treatments. Scale bar, 100 μm.
